# Augmented, Mixed, and Virtual Reality-Based Head-Mounted Devices for Medical Education: Systematic Review

**DOI:** 10.2196/29080

**Published:** 2021-07-08

**Authors:** Sandra Barteit, Lucia Lanfermann, Till Bärnighausen, Florian Neuhann, Claudia Beiersmann

**Affiliations:** 1 Heidelberg Institute of Global Health Heidelberg Germany; 2 Department of Global Health and Population Harvard TH Chan School of Public Health Boston, MA United States; 3 Africa Health Research Institute KwaZulu-Natal South Africa; 4 School of Medicine and Clinical Sciences Levy Mwanawasa Medical University Lusaka Zambia

**Keywords:** virtual reality, augmented reality, global health, income-limited countries, medical education

## Abstract

**Background:**

Augmented reality (AR), mixed reality (MR), and virtual reality (VR), realized as head-mounted devices (HMDs), may open up new ways of teaching medical content for low-resource settings. The advantages are that HMDs enable repeated practice without adverse effects on the patient in various medical disciplines; may introduce new ways to learn complex medical content; and may alleviate financial, ethical, and supervisory constraints on the use of traditional medical learning materials, like cadavers and other skills lab equipment.

**Objective:**

We examine the effectiveness of AR, MR, and VR HMDs for medical education, whereby we aim to incorporate a global health perspective comprising low- and middle-income countries (LMICs).

**Methods:**

We conducted a systematic review according to PRISMA (Preferred Reporting Items for Systematic Reviews and Meta-Analysis) and Cochrane guidelines. Seven medical databases (PubMed, Cochrane Library, Web of Science, Science Direct, PsycINFO, Education Resources Information Centre, and Google Scholar) were searched for peer-reviewed publications from January 1, 2014, to May 31, 2019. An extensive search was carried out to examine relevant literature guided by three concepts of extended reality (XR), which comprises the concepts of AR, MR, and VR, and the concepts of medicine and education. It included health professionals who took part in an HMD intervention that was compared to another teaching or learning method and evaluated with regard to its effectiveness. Quality and risk of bias were assessed with the Medical Education Research Study Quality Instrument, the Newcastle-Ottawa Scale-Education, and A Cochrane Risk of Bias Assessment Tool for Non-Randomized Studies of Interventions. We extracted relevant data and aggregated the data according to the main outcomes of this review (knowledge, skills, and XR HMD).

**Results:**

A total of 27 studies comprising 956 study participants were included. The participants included all types of health care professionals, especially medical students (n=573, 59.9%) and residents (n=289, 30.2%). AR and VR implemented with HMDs were most often used for training in the fields of surgery (n=13, 48%) and anatomy (n=4, 15%). A range of study designs were used, and quantitative methods were clearly dominant (n=21, 78%). Training with AR- and VR-based HMDs was perceived as salient, motivating, and engaging. In the majority of studies (n=17, 63%), HMD-based interventions were found to be effective. A small number of included studies (n=4, 15%) indicated that HMDs were effective for certain aspects of medical skills and knowledge learning and training, while other studies suggested that HMDs were only viable as an additional teaching tool (n=4, 15%). Only 2 (7%) studies found no effectiveness in the use of HMDs.

**Conclusions:**

The majority of included studies suggested that XR-based HMDs have beneficial effects for medical education, whereby only a minority of studies were from LMICs. Nevertheless, as most studies showed at least noninferior results when compared to conventional teaching and training, the results of this review suggest applicability and potential effectiveness in LMICs. Overall, users demonstrated greater enthusiasm and enjoyment in learning with XR-based HMDs. It has to be noted that many HMD-based interventions were small-scale and conducted as short-term pilots. To generate relevant evidence in the future, it is key to rigorously evaluate XR-based HMDs with AR and VR implementations, particularly in LMICs, to better understand the strengths and shortcomings of HMDs for medical education.

## Introduction

Augmented reality (AR), mixed reality (MR), and virtual reality (VR)–based technologies open novel ways of teaching and training for medical education, as they allow for immersive experiences that may foster the teaching and learning of complex medical contents. Especially so-called head-mounted devices (HMDs), most often realized as a headset or glasses, seem to be advantageous and adequate to low- and middle-income countries (LMICs) based on their versatile, low-price, and mobile nature [1,2]. Technologies like HMDs make learning content more accessible and engaging, whereas for educators, it broadens their educational impact beyond the classroom and face-to-face teaching [3,4]. Quality education is key to improve health outcomes for all [5], especially in low-resource settings where there is a dire need to strengthen the health workforce [6], particularly today, as health professionals must acquire a great deal of skills and know-how to become competent practitioners [7]. HMDs can potentially be a catalyst for improving educational efforts by increasing the effectiveness of existing medical training programs, as AR-, MR-, and VR-based HMDs enable repeated practice without adverse effects on the patient in various medical disciplines; may introduce new immersive ways to learn complex medical content; and may alleviate financial, ethical, and supervisory constraints on the use of traditional medical learning materials like cadavers and other skills lab equipment [8-11]. Moreover, disruptive technologies such as HMDs can not only help to learn but also prepare medical learners for a highly technologically advanced workplace [12]. Therefore, HMDs hold the promise to be a potential driver in strengthening health systems and the health workforce, which has been key to increasing global life expectancy in recent years [13]. Particularly, LMICs face health worker shortages, skewed distribution of health professionals toward urban areas, and limitations in skill sets and training that do not aptly address the population’s real health needs [13,14]. Regions in Africa and Asia still have alarmingly high health worker shortages [10] despite the Sustainable Development Goals (SDGs) calling for a substantial increase in the recruitment, development, training, and retention of the health workforce in income-limited countries (SDG goal 3C) [15]. To successfully achieve the SDGs for 2030, digital technologies may be a key element, as they bear the potential to enhance health professional performance and training in a rapid and cost-effective way [1,2]. In addition, digital technologies are versatile and are well able today to reflect the varied training needs of health professionals covering a broad field of teaching and training needs, clinical competencies, and skills such as therapeutic and diagnostic skills and communication skills. Nowadays, technologies provide an experience close to reality, without putting the patient at risk during training. Technologies provide a quality standard of technical medical skills, as they are scalable and repeatable until skills are fit for practice. In particular, HMDs are versatile in their use compared to specialized individual simulators already used for medical training and available at low prices, and for providing increased learning space mobility—features particularly valuable in low-resource contexts [1,2,8,9].

Currently, there is a lack of insights on HMDs particularly in LMICs, as most reviews on HMDs have focused on high-income countries, on AR for medical education, or on AR and VR but not in the context of health [16-20]. This systematic review took a global perspective. Technologies are constantly evolving and there is a need for obtaining an overview of current trends in a global context. No other review was found that had considered HMDs for global health professional training considering a recent time frame.

The main objective of this systematic review is to screen the current literature evaluating HMDs using AR, MR, and VR for medical education, and to elucidate the effectiveness of HMDs for medical education in a global context, particularly with regards to LMICs. Two main research questions guided the systematic review: (1) what is the effectiveness of using HMDs for medical education, specifically for knowledge and skills, and (2) what are the strengths and weaknesses of HMDs in medical education?

## Methods

This systematic review was conducted according to the Cochrane Collaboration Handbook for systematic reviews [21] and reported according to PRISMA (Preferred Reporting Items for Systematic Reviews and Meta-Analyses; see Multimedia Appendix 1) [22]. There was no review protocol published and the review was not registered.

### Data Source, Search Strategy, and Inclusion and Exclusion Criteria

Seven medical and educational databases for peer-reviewed literature were searched comprising PubMed, Cochrane Library, Web of Science, Science Direct, PsycINFO, Education Resources Information Centre, and Google Scholar (200 first results were extracted, according to Haddaway et al [23]). Gray literature databases that were searched comprised WorldCat (45 results were used from each of the three search concepts *XR*, *medicine*, and *education*) and Global Index Medicus.

In addition, reference lists of selected articles have been hand-searched and included if inclusion and exclusion criteria were met. As relevant HMD technology was first commercially introduced in 2014 [24], publications were only included if published after January 1, 2014, until the end of the study period, which was May 31, 2019, and were restricted to the English language. Surveys, editorials, and conference papers were excluded, as well as literature that had no abstract or full-text available.

**Figure 1:**
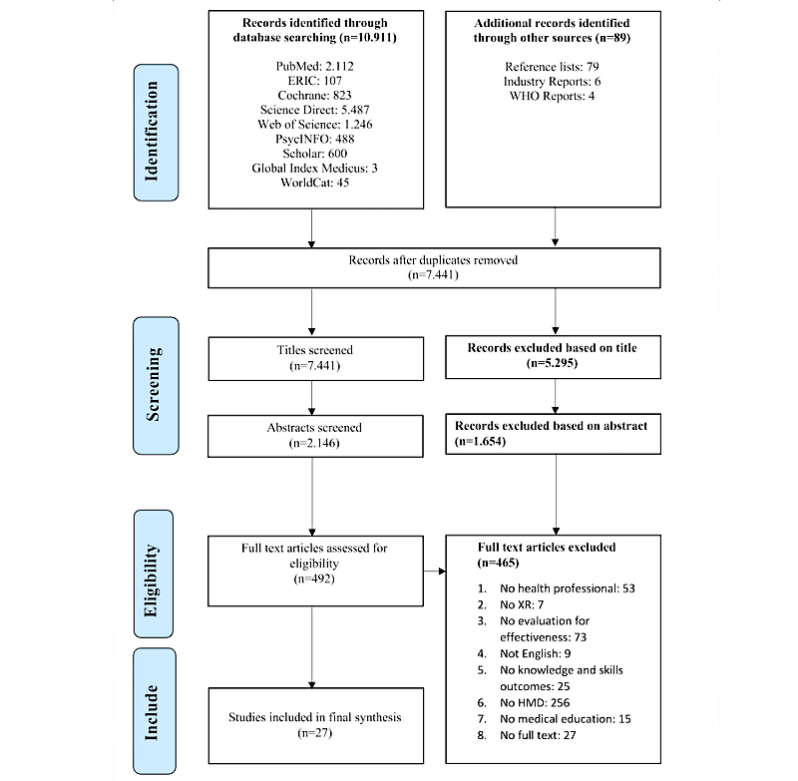
PRISMA (Preferred Reporting Items for Systematic Reviews and Meta-Analyses) flowchart for systematic reviews. ERIC: Education Resources Information Centre; HMD: head-mounted display; WHO: World Health Organization; XR: extended reality.

The PICOS (population, intervention, comparison, outcome, study design) framework guided the inclusion and exclusion criteria of this study [25] (see Table 1). To ensure coverage of all relevant literature to this rather novel topic, search terms were compiled comprehensively and were grouped into the three search concepts of *extended reality* (XR; which subsumes the concepts of VR, AR, and MR), *medicine*, and *education*. The following search terms or keywords were used alone or in combination: *virtual reality*, *augmented reality*, *mixed reality*, *medical*, *health*, *clinical*, *education*, *teaching*, *training*, and *learning* (see Multimedia Appendix 2 for a detailed overview of the search strategy). The PRISMA flowchart is shown in Figure 1 depicting the screening process.

**Table 1:** PICOS (population, intervention, comparison, outcome, study design) framework (adapted from Methley et al [25]).

Framework	Description	Inclusion	Exclusion
Population	Health professionals who received medical education	Health professionals, nursing and midwifery professionals, modern health associate professionals, nursing and midwifery associate professionals	Health management and support workers who are not in health service provisioning
Intervention	Head-mounted displays based on virtual reality, augmented reality, or mixed reality	Head-mounted displays of all kinds that include glasses or goggles	Devices that are not head-mounted displays
Comparison	Modern vs traditional methods for medical education to evaluate effectiveness of XR^a^ tools	Books, pen and paper, chalkboard, face-to-face teaching, traditional lectures	No evaluation of the effectiveness of XR devices
Outcome	Improved or not improved learning outcome	Concrete learning outcome/evaluation of effectiveness in learning	No concrete outcome
Study	Literature in English, published between Jan 1 to May 31, 2019	Literature as identified via the search strategy	Literature reviews, meta-analyses, opinion papers; non-English literature; literature published before Jan 1, 2014, and after May 31, 2019

^a^XR: extended reality.

### Data Extraction and Study Quality Assessment

Articles were independently screened by two researchers using the eligibility criteria of the PICOS framework. Data were extracted as recommended by the Cochrane Handbook [21] (for detailed data extraction table see Multimedia Appendix 3). Any disagreements were resolved by discussion between the two screening authors. Population characteristics were derived from the studies, including author, year, place, journal, country of study, study design, evaluation methods, number of evaluation methods, type of data analysis, effectiveness, type of study population, medical discipline of study, type of XR, type of HMD, type of learning, study duration, and references.

Quality and risk of bias was assessed with the Medical Education Research Study Quality Instrument (MERSQI), the Newcastle-Ottawa Scale-Education (NOS-E), and “A Cochrane Risk of Bias Assessment Tool for Non-Randomized Studies” (ACROBAT-NRSI) [26-28].

### Synthesis Method

Data synthesis is reported according to the SWiM (Synthesis Without Meta-analysis) reporting guideline [29]. The research question guided the synthesis groupings, and we focused on the effect of XR HMDs on knowledge and skills gained in relation to the HMD devices used. Furthermore, we reflected each synthesis group according to their medical specialties, as we thought that each medical specialty is different in its focus on knowledge or skills training. No standardization metric was applied. The synthesis method was to extract relevant sections of the studies with regard to knowledge and skills gained, as well as the specific XR HMD. Overall, the risk of bias assessment of nonrandomized study designs showed no critical results (see Multimedia Appendix 4 for details). Accordingly, the 27 included studies were synthesized with equal weight. We did not restrict the study design. Hence, we did not conduct a meta-analysis, as studies were quite heterogenous, and the included studies reported different quantities and qualities about knowledge and skills. Effectiveness was synthesized according to the respective study reports. The tables aggregate information about the study characteristics and focal areas of this review (knowledge, skills, XR HMD).

## Results

### Study Characteristics

A total of 27 studies was included in the review: 17 (63%) VR studies, 7 (26%) AR studies, 2 (7%) MR-focused studies, and 1 (4%) VR and AR study (see Table 2 for study details). Although 24 (89%) studies used only a single HMD, 3 (11%) studies compared two HMDs. All studies were in an academic or hospital setting and mostly compared HMDs to conventional face-to-face training methods [2,30-41]. The included studies used an HMD worn on the head.

Included studies were categorized according to three levels of knowledge (adapted and modified from Górski et al [42], see Multimedia Appendix 5): (1) theoretical knowledge (eg, anatomical atlases and preoperative planning), (2) practical skills (eg, operation trainings and surgical simulators), and (3) attitudes (eg, self-confidence, communication skills, and patient-centeredness).

Overall, the included studies (N=27) comprised a study population of 956 study participants. Sample sizes ranged from 1 participant to 178 participants, with a mean of 35 participants (see Multimedia Appendix 6 for details of the number and type of study participants).

The medical procedures described across studies varied widely. Studies in surgery included training in neurosurgery [43], gastrectomy [2], total hip arthroplasty [35,37], laparoscopy [44], dental surgery [32,45], surgical ophthalmoscopy [33], peg transfer practice [46], or surgical knot training [47] and central line and catheter insertion [48,49]. Anatomy teaching was covered in 4 (15%) studies, 3 of which involved 3D learning structures in neuroanatomy [39,50,51], such as training on the brain cerebrum. Luursema et al [34] focused on the effect of visual ability on anatomical understanding. Ferrandini Price et al [38] trained emergency medicine with a “triage for a mass casualty incident,” Rai et al [31] focused on ophthalmoscopy with “binocular indirect ophthalmoscopy,” Siff and Mehta [52] evaluated “an interactive holographic curriculum” for gynecology training, and Bing et al [53] looked into cervical cancer surgery in Zambia. In the field of urology, Butt et al [40] conducted catheterization. Digital slides were analyzed in the field of pathology [54]; dental implants in dentistry; [45] and, in the field of geriatrics, Dyer et al [55] concentrated on neurodegenerative diseases.

**Table 2:** Characteristics of included studies.

Study characteristics				Studies (N=27), n (%)
**Year of publication**				
	2014-2015		4 (15)	
	2016-2017		9 (33)	
	2018-2019		14 (52)	
**Country classification by income-level**				
	Low-income country		0 (0)	
	Middle-income country		2 (7)	
	High-income country		25 (93)	
**Country of study**				
	US		10 (37)	
	Canada		3 (11)	
	UK		3 (11)	
	Australia		2 (7)	
	Germany		2 (7)	
	France		1 (4)	
	Ireland		1 (4)	
	Spain		1 (4)	
	Netherlands		1 (4)	
	China		1 (4)	
	Taiwan		1 (4)	
	Zambia		1 (4)	
**Reported study design**				
	Quantitative		21 (78)	
	Qualitative		5 (19)	
	Mixed methods		1 (4)	
**Evaluation methods**				
	Skills tests		18 (67)	
	Questionnaires		16 (59)	
	Recordings		6 (22)	
	Knowledge tests		5 (19)	
	Surveys		4 (15)	
	Observation		4 (15)	
	Self-assessment		3 (11)	
	Others		5 (19)	
**Number of evaluation methods used**				
	One method		16 (59)	
	Two methods		9 (33)	
	Three methods		2 (7)	
	More than three methods		0 (0)	
**Data analysis conducted in publication**				
	Inferential statistics		15 (56)	
	Descriptive statistics		10 (37)	
	Qualitative analysis		1 (4)	
	No analysis identified		1 (4)	
**Self-concluded effectiveness**				
	Effective		17 (63)	
	Partly effective		4 (15)	
	Useful only as additional tool		4 (15)	
	No proven effectiveness		2 (7)	
**Study population^a^**				
	Students		10 (37)	
	Residents		8 (30)	
	Physicians/nurses		3 (11)	
	Mixed training levels		6 (22)	
**Medical discipline**				
	Surgery		13 (48)	
	Anatomy		4 (15)	
	Gynecology		2 (7)	
	Emergency medicine		2 (7)	
	Ophthalmology		2 (7)	
	Urology		1 (4)	
	Pathology		1 (4)	
	Geriatrics		1 (4)	
	Dentistry		1 (4)	
**Mode of XR^b^ used**				
	VR^c^		17 (63)	
	AR^d^		7 (26)	
	MR^e^		2 (7)	
	Combined information		1 (4)	
**Type of head-mounted display^f^ (model and type of XR)**				
	Oculus Rift (Consumer V1/DK2; VR)		8 (30)	
	HTC Vive (2016; VR)		4 (15)	
	Samsung Gear VR (not specified; VR)		4 (15)	
	MS HoloLens (Development Ed; MR)		3 (11)	
	Eyesi Indirect System Simulator (Version 1.1.3; AR)		2 (7)	
	Google Glass (Trial Version; AR)		2 (7)	
	No information on brand		2 (7)	
	Brother AirScouter (WD-200B)		1 (4)	
	Daydream View Headset (Not specified; VR)		1 (4)	
	Epson Moverio (BT-200; AR)		1 (4)	
	Sony HMZ (T1 3D Viewer; VR)		1 (4)	
**Type of learning and medical discipline**				
	**Practical skills**		18 (67)	
		Surgery	11 (41)	
		Emergency medicine	2 (7)	
		Ophthalmology	2 (7)	
		Dentistry	1 (4)	
		Urology	1 (4)	
		Gynecology	1 (4)	
	**Theoretical knowledge**		7 (26)	
		Anatomy	4 (15)	
		Surgery	1 (4)	
		Pathology	1 (4)	
		Gynecology	1 (4)	
	**Attitudes**		2 (7)	
		Geriatrics	1 (4)	
		Surgery	1 (4)	
**Duration of Intervention**				
	<1 month		21 (78)	
	1 month to 6 months		3 (11)	
	7-12 months		1 (4)	
	1 year to 2 years		1 (4)	
	>2 years		1 (4)	

^a^Residents were medical doctors in specialized training after completing medical school, under supervision of an experienced doctor. Physicians or nurses were professionals with several years of accredited experience in their field. Mixed training levels indicate that participants of two or more distinct educational levels were part of the study, such as residents and students combined.

^b^XR: extended reality.

^c^VR: virtual reality.

^d^AR: augmented reality.

^e^MR: mixed reality.

^f^Multiple types of head-mounted displays may have been used within a study.

### Effectiveness of Knowledge, Skills, and Attitudes

In most of the 27 studies, skills were taught with HMDs in the field of surgery (n=18, 67%). Some studies (n=7, 26%) focused on knowledge transfer, mainly in the field of anatomy, and a few studies (n=2, 7%) trained on perceptions and self-confidence. HMDs varied across studies by brand, model, functionality, and type of XR (see Multimedia Appendix 7 for details on reported effectiveness).

### Skills

Out of the 27 studies, 18 (67%) were identified that focused on skills outcomes [2,30,31,33,35-38,40,41,44-49,53,56].

#### Surgery (11 Studies)

Barré et al [2] explored a VR-based scenario to train medical professionals on sleeve gastrectomy, which study participants described as “realistic and useful to learn surgery” (device: HTC Vive). Sensors for the VR-based scenario were attached to real-life surgical instruments for navigation in a virtually created operating room. The authors found a reduction in cognitive effort and a decrease in stress during prolonged periods of standing during surgical practice [2]. Huang et al [48] focused on “AR simulation of venous catheters” (device: Brother AirScouter), in which AR glasses were used to display instructions on procedural steps of catheter insertion in the form of a digital overlay of information to participants during the medical process. Using AR HMDs was perceived as useful for skills transfer in the operating room [48]. Yoganathan et al [47] found that 360° videos of surgical knot training produced major advantages, as they improved the ability to tie knots during surgery even as stand-alone learning (device: unspecified). Hooper et al [37] taught hip arthroplasty, but the results were unclear as to the impact on medical knowledge (device: Oculus Rift CV1). The authors reported that if the VR training was conducted right before the actual surgery, it may have a positive impact on technical abilities and acknowledged that the impact of VR for surgery would significantly increase in the years to come. Logishetty et al [35] found that the quality of hip surgery performed through AR did not differ from that experienced by in-person training with a physician (device: MS HoloLens). Nonetheless, the authors still perceived AR as a valuable tool for other surgical procedures related to arthroplasty. Peden et al [33] tested HMDs for the learning of suture skills by medical students, whereby standard face-to-face instruction compared to HMDs showed similar skills outcomes (device: Google Glass). Qin et al [46] compared simulators and a variety of XR devices for peg transfer training (devices: HTC Vive, Samsung Gear). Overall, medical simulation systems contributed to a more immersive and successful training environment for peg transfer [46]. Rochlen et al [49] evaluated the usability and feasibility of AR technology for needle insertion for central venous catheter placement for medical students (device: Google Glass). The authors found that AR technology may constitute an important addition to medical skills training.

Harrington et al [56] discovered that surgeons were more attentive when training in 360° instead of in 2D environments for learning laparoscopic cholecystectomy (device: Samsung Gear VR). The study participants, which were preclinical undergraduate students, found the 360° learning beneficial and entertaining, whereby no significant difference in information retention was found between 360° learning and 2D videos [56]. Huber et al [44] investigated experiences of learning laparoscopic skills (peg transfer, cholecystectomy) with a VR-based HMD (device: HTC Vive) compared to a simulator and found that the use of training with HMD was feasible and that participants were excited about the immersion provided by the HMD. Accuracy scores between the two comparison groups were equal, so the outcome of laparoscopic performance was noninferior [44]. Wu et al [30] tested the feasibility of ultrasound procedures (ultrasound-guided central line procedure) with two groups: one group with the AR-based HMD (device: Google Glass) and the other group using traditional ultrasound [30]. The AR-based HMD projected a digital layer at the corner of the glasses to see whether physicians could increase their focus on the procedure. Overall, the authors found that the Google Glass could be effective in ultrasound training, with the only caveat that study participants needed more time being familiarized with the technology, as well as “more needle redirections, but less head movements” [30].

#### Emergency Medicine (2 Studies)

Azimi et al [41] evaluated whether nursing students and novice learners could improve the learning of the emergency medical procedure of needle chest decompression virtually using an HMD (device: MS HoloLens) [41]. There were no adverse effects of nurses’ emergency training using HMDs, and participants improved in terms of frequency of training and enthusiasm, and recalled and demonstrated proficiency in their training days later [41]. In an emergency scenario with VR HMDs, Ferrandini Price et al [38] tested the stress of study participants of “basic triage in a mass accident event” and found that VR cannot yet replace a clinical simulation. Therefore, they recommended VR as a complementing training method (device: Samsung Gear VR).

#### Ophthalmology (2 Studies)

Leitritz et al [36] found that inexperienced students practice ophthalmoscopic examination better after HMD training (device: ARO). Rai et al [31] compared AR to face-to-face teaching in the field of ophthalmoscopy and found that AR simulation may be superior for skills learning, especially for novice ophthalmology students (device: EyeSI BIO simulator with headpiece).

#### Dentistry (1 Study)

Lin et al [45] tested an AR-based dental implant placement system (device: Sony HMS-T1) and evaluated the precision of the virtually planned versus the actual prepared implant position. The integration of the AR technology considerably reduced the deviation of placing the implant from planned position, and the accuracy of computer-aided implant surgery increased [45].

#### Urology (1 Study)

Butt et al [40] reviewed VR systems for “urinary catheterization” and found that undergraduate nursing students were enthusiastic about applying it to practical scenarios (device: Samsung Gear).

#### Gynecology (1 Study)

Bing et al [53] replicated hysterectomy training (removal of uterus) in a virtual 3D environment for Zambian surgery trainees (device: Oculus Rift). The authors concluded that “affordable VR might have the potential to enhance access to cancer treatment globally” [53].

### Knowledge

Out of the 27 studies, 7 (26%) studies evaluated the outcome of knowledge with regard to XR interventions [34,39,43,50-52,54].

#### Anatomy (4 Studies)

Moro et al [50] evaluated the acquisition of anatomical knowledge (spine anatomy) and compared two different learning modes: desktop-based (device: Oculus Rift) and mobile-based (device: Gear VR). Both groups performed similarly on the knowledge test, although a number of study participants experienced fuzzy vision and malaise using VR. Stepan et al [51] identified neuroanatomical test scores of participants using HMDs (device: Oculus Rift) compared to participants who practiced with conventional anatomy books. The VR-based training led to more engagement, learner motivation, and enjoyment, although there was no difference in exam performance between the two groups. Ekstrand et al [39], evaluated in a randomized controlled study the benefits of VR-based HMD neuroanatomy training and compared it to learning with a paper-based 15-page booklet focusing on anatomy training for medical students. Ekstrand et al [39] mentioned that the HTC Vive in neuroanatomy training can be used as an additional tool to increase knowledge gain but that the VR group did not surpass the control group with respect to learning outcomes. They found similar knowledge test results for VR and learning from books [39]. Luursema et al [34] examined the usability of VR-based HMDs for anatomy learning (cognitive load and problem-solving strategies) for users with respect to stereoptic depth. They concluded that it was not evident whether depth perception advanced or impeded the uptake of anatomical knowledge. The Luursema et al [34] study stated that digital interventions neither enhanced nor impeded knowledge outcomes in anatomy.

#### Surgery (1 Study)

Bairamian et al [43] compared a 3D-printed model to VR-based HMD angiogram models (device: Google Daydream HMD in connection with a smartphone). The HMD offered better resolution and zoom capabilities for study participants (neurosurgical trainees, neurosurgeons), but the 3D-printed model offered better depth perception and manipulation opportunities. The authors concluded that VR-based HMD angiogram may be a viable alternative to 3D-printed models, with untapped educational potential [43].

#### Pathology (1 Study)

Farahani et al [54] evaluated the feasibility of using VR-based HMDs for reading digital pathology slides of lymph nodes (device: Oculus Rift). Study participants (pathologists) reported that VR pathology slides were limited in their resolution and that they faced difficulties navigating the VR device. Overall, study participants were able to produce accurate diagnoses, and high diagnoses concordance was reached compared between the VR-based HMD and traditional slide system [54].

#### Gynecology (1 Study)

Siff and Mehta [52] introduced an interactive holographic training module for teaching urogynecologic surgical anatomy (device: MS HoloLens), which involved holograms of female organs, livestreaming of surgical videos, and 3D-projected organs to enhance structural understanding (ligament suspension, sacrospinous ligament fixation). Siff and Mehta [52] observed that the interactive holographic mode of learning was effective in the acquisition of knowledge for surgical anatomy. Study participants ranked the AR-based training as much better when compared to conventional training [52].

### Attitudes

Out of the total 27 studies, 2 (7%) studies [32,55] evaluated the effects of HMD training on specific attitudes of health professionals. Dyer et al [55] investigated whether the understanding of neurodegenerative diseases and their impact on patients could be made more transparent to medical students by training with a VR-based HMD (device: Oculus Rift). The results revealed that the impact of simulation on the attitudes of participants was significant [55]. Pulijala et al [32] focused on the impact of “VR surgery on the self-confidence of surgical residents.” They found that the “self-esteem of participants could be increased” [32] when training with the HMD (device: Oculus Rift). This was especially true for participants with little clinical experience. Both interventions were evaluated as effective, since understanding of diseases and self-confidence in surgery increased. The effect on self-confidence was “especially high for inexperienced physicians” [32].

### Evaluation Methods

Of the 27 studies, the most common evaluation methods were practical skills tests (n=18, 67%), followed by questionnaires (n=16, 59%), video recordings of procedures (n=6, 22%), knowledge tests (n=5, 19%), surveys (n=4, 15%), observations (n=4, 15%), self-assessments (n=3, 11%), and others (n=5, 19%).

Practical skills tests comprised in some cases of observing study participants when repeating learned procedures or repeating practical skills. Questionnaires included general questionnaires, questionnaires combined with follow-up assessments, and Likert scales. Generally, authors used multiple questionnaires throughout the interventions. Videos were generally recorded during practice sessions of medical trainings and were later evaluated. Bing et al [53] measured how participants moved during the simulation and how much time was spent to fulfill the task. The surgeons could verify their performance scores that were recorded by the simulator. Knowledge and skills were usually assessed prior to and post intervention. Surveys followed the *Validation of Instructional Materials Motivation Survey* [57] or the *System Usability Survey* for the measurement of participant perceptions on usefulness [40]. Participant self-assessment was also evaluated, in which authors asked students to assess their own performance, as self-written evaluation text or self-assessment questionnaire [2,52,53]. Other evaluation methods included using students’ drawings of the nerve head to assess diagnostic capabilities [36], saliva samples to elucidate stress levels during training [38], the use of dental computer tomography images to measure the differences between implant locations [45], and examination of the precision of acetabular placement [35].

## Discussion

### General Aspects

The results showed that HMDs are at least comparable to traditional methods of medical education and beneficial in terms of increasing students’ motivation for learning (see Multimedia Appendix 8 for an overview of benefits, shortcomings, and recommendations described within included studies). HMDs allow for repeated use of difficult training scenarios in an immersive and realistic environment, such as emergency procedures or rare complications during surgery. The studies found benefits and shortcomings of HMDs. Studies based their findings on improving and enhancing HMDs.

XR-based HMDs are currently dominantly used in high-income countries; only 2 [46,53] studies were conducted in LMICs. Nevertheless, HMDs may be particularly beneficial in a low-resource context to provide training for direly needed health care workers based on their versatile, mobile, and immersive nature. Bing et al [53] implemented and evaluated HMDs for surgical training in Zambia, which was found to be effective. The adoption of XR-based HMDs in other medical settings may most likely increase in the next years and may foster medical teaching and training especially in settings where there is a need for time-effective and cost-effective education [58]. Particularly, recent developments like the HMD Oculus Quest seem to be particularly promising for LMIC contexts. The potential for XR-based HMDs in other countries and settings, particularly in LMICs, need to be studied further. In addition, long-term effects of using HMDs on learners’ knowledge and skills in various medical education settings and the integration into medical curricula needs to be further researched. Further technical advancements are needed, and traditional methods of education are not to be ignored.

In the following sections, the Discussion is structured according to the research questions of this review: What is the effectiveness of using HMDs for medical education, specifically for knowledge and skills, and what are the strengths and weaknesses of HMDs in medical education?

### Effectiveness

The studies in this review generally categorized an intervention as effective if the majority of the study population achieved higher scores in tests (pre-posttest, exercises) or participant observations as compared to traditional instructional approaches, such as books and analogue surgery or ultrasound procedures.

Not all studies reported effective outcomes for the use of HMDs in medical education. Some studies described the disadvantages of HMDs, such as motion sickness and nausea, technical problems, and stress. A systematic review underlined that these disadvantages may impede learning and training [9]. It is unclear if symptoms of motion sickness and nausea are related to beginners’ attempts to become familiar with the technology or if these persist long-term and may potentially impede learning or education. A study has found that women more often are faced with motion sickness using VR devices and that stabilization of the users’ body may alleviate symptoms [59]. In addition, effects may differ as AR devices combine real and virtual environments, which should reduce the experienced adverse health effects in VR applications, such as blurred vision, disorientation, and cybersickness. These adverse effects of individuals using HMDs may vary depending on the device. As, for example, AR devices combine real and virtual environments, they seem to mitigate negative health effects such as blurred vision, disorientation, and cybersickness [7].

It has to be taken into account that technology acceptance for XR-based HMDs may differ between individual learners, as inexperienced users may require more time and effort using HMDs for educational purposes.

In addition, realistic feedback was at times not implemented. For instance, “surgical errors that occurred in virtual training were not followed by complications such as simulated patient bleeding or variations in anatomy” [53]. Contextual factors such as sizes, sounds, and functionalities of instruments of virtual operating rooms need to be extremely precise and realistic. Otherwise, there is the potential of erroneous learning and training [8]. Nevertheless, with the continuous development of technologically sophisticated learning tools, more advancements become available that may supplement XR-based HMDs with audio and visual information [7].

### Skills

The progression from knowledge to skills follows four levels of competences as per Miller’s Pyramid of Professional Competence [60]. Particularly, the *see one, do one, teach one, and simulate one* concept in line with Miller’s Pyramid underlines the potential of XR-based HMDs, which can implement this concept for medical teaching and skills training. Particularly, as students have limited time to learn with cadavers, if at all, they are required to supplement their anatomical knowledge through self-directed study. This material is frequently presented in the form of 2D supplementary resources such as lecture slides, textbooks, and flashcards [7]. HMDs, like other disruptive technologies, encourage students to be active and self-directed learners who set their own learning pace through hands-on experiences. Active learning has shown to lead to improved educational outcomes such as increased learning retention [61]. Through the use of XR-based HMDs, learners may gain a more realistic understanding of medical concepts [19]. Learners training with HMDs were shown to improve practical skills, reduce stress, and gain self-confidence for the actual surgery [32,52]. Furthermore, the high level of experience needed in the operating room can be easily and repeatedly gained with XR-based HMDs [8,16]. Our review highlighted possibilities of repeatedly practicing surgical operations such as hysterectomy, laparoscopy, or total hip arthroscopy via HMDs. In this review, HMDs were mostly used in the fields of surgery and anatomy with positive skill outcomes. The main benefits of studies have shown decreased surgical error rates, cost-effectiveness, and improved knowledge. The predominance of surgery and anatomy may be that both have a long-standing history of simulation training beginning in the late 1980s with simple simulation [62]. In ophthalmology, examining the virtual retina and gaze of a virtual patient increases practice time, as normally, practice is with the real pupils of a patient’s eye [31,36]. Resultantly, less patients need to participate in training sessions in ophthalmology [36]. A potential application that seems adequate for low-resource contexts could be HMD-based training for cataract surgery, as cataracts are still quite common in these settings [63].

During emergencies, physicians work under increased intensity while simultaneously working at reduced capacity due to increased stress. XR-based training scenarios can be effective in helping clinicians better understand the situations they need to be prepared for [8,64].

### Knowledge

Some studies in this review pointed out that 3D models of human bodies significantly improved learning outcomes, with HMDs providing 360° views, as new structures and concepts are processed to enhance overall comprehension [65]. “Threshold concepts” are crucial to understand practical applications, such as with anatomy [65]. Without knowledge of anatomical structures, students will not be able to excel in surgery. The findings in this review suggest that XR-based HMDs set the hurdle lower for “threshold concepts” because students immerse themselves in physical structures, which increases understanding and later information retrieval during actual operations. In addition, using HMDs to view 3D brain structures appears to be more motivating and engaging than traditional methods such as books [8,39,51,66]. The lack of spatial understanding and imagination when reading traditional books might increase this phenomenon [39]. HMDs enable the understanding of complex organ structures [66]. There has been a significant increase in volume of medical information expected of students in modern times, and they are moving into technology-enhanced resources to increase learner engagement and authenticity [11].

Despite higher motivation and enjoyment for learning anatomy with HMDs, there was no significant difference in knowledge acquisition when compared to learning from textbooks [39,51]. This is similar to other reports [11], and similar to other XR applications, such as for patient treatment where studies that did examine efficacy demonstrated preliminary evidence of similar effectiveness or more effectiveness of XR interventions than their selected conventional therapy [67]. Ultimately, increased motivation to study and understand 3D structural complexities is an important aspect [51].

The duration of the various XR HMD study sessions has yet to be examined in detail in the numerous studies that have been done. Some may find it more pleasant to spend longer or shorter periods training with XR HMDs to gain the greatest possible knowledge [7]. The fact that there is no difference in learning outcomes when using XR-based HMDs is encouraging and validates the possibilities for incorporating these innovative technologies into medical education [11]. Likely, the most effective method to teach anatomy, for example, may be to combine multiple resources in addition to XR-based HMDs like plastic models, dissections, and learning software [7]. Particularly in LMICs, XR-based HMDs may be able to strengthen and scale interactive learning and training in anatomy and other surgical skills.

### Attitudes

Attitudes are often neglected in medical education to the detriment of patients [68]. Communication and problem-solving skills as well as empathy are vital for patient care. Studies of this review showed that not only diagnostic and therapeutic skills can be trained, but also attitudes. For example, in the field of geriatrics, XR-based HMD training was shown to foster empathy for older adult patients [55]. The simulation was a scenario in which the user was an older person with an age-related disease such as Alzheimer disease. Being immersed in this surrounding provided a realistic point-of-view into older patients’ perceptions. In addition, physicians were successfully trained on communication skills while interacting with patients via VR-based avatars [69]. Through XR-based virtual patients, difficult conversations can be simulated [70]. In this way, communication and other soft skills may be trained more effectively and detailed for handling challenging patient situations. HMDs can be used for both diagnostic and therapeutic training as well as for communication skills and attitudes.

### Evaluation Methods

Most included studies evaluated the effectiveness of HMDs (ie, through pre- and postknowledge tests, practical exercises, or self-assessments). The term effectiveness is used in many different contexts with varying definitions [71], consisting of several components: feasibility, cost, safety, and applicability to specific contexts [72]. A successful technological implementation and evaluation considers multiple levels: the individual learner, the learning environment, the context of the learning implementation, the technological environment, and the pedagogics involved in the learning implementation [73]. In studies, the criteria determining the effectiveness or ineffectiveness of an intervention was not based on a unified definition. Whereas some studies provided more information on HMD costs, others focused more on ways that XR-based HMDs can be used across various medical disciplines. Adhering to a standardized framework for implementation and evaluation of XR-based HMDs is key to generating good and comparable evidence that can guide digital health implementations. To this end, first endeavors seem to be on their way to improve methodological quality. The Virtual Reality Clinical Outcomes Research Experts (VR-CORE) international working group compiled a three-part framework for best practices in developing and testing VR treatments to improve patient outcomes. This framework targets the development of high quality, effective, and safe VR treatments [74]. Similarly, a framework would be needed to guide XR-based interventions for medical education. For the evaluation of XR-based interventions, the World Health Organization guide on monitoring and evaluating digital health interventions may already be used as a guide [58].

### Strengths and Limitations

Study quality and bias were evaluated using three assessment tools (MERSQI, NOS-E, ACROBAT-NRSI). Most studies in this systematic review had relatively high quality scores for study design, sampling response rates, and types of data. This might be due to a large number of randomized controlled trials included in this review. This paper followed well-established methods of conducting and reporting systematic reviews [21,22].

The methodology of this review had limitations. Participants were restricted to medical professionals. In addition, solely English publications were included, which could have led to the exclusion of relevant articles in other languages. Only HMDs were included, even though other devices that use XR in medical education exist (eg, magic mirrors, large simulators, or serious games). Hence, the focus of this review was on mobile solutions in the field. Not all gray literature was screened, which could have also led to the exclusion of suitable papers. One further limitation is that we did not consider the costs of developing XR content, which is of particular relevance for XR-based applications in LMICs. Future studies should take into consideration effort and costs of developing XR-based content to provide a better basis for decision-making.

Although various studies have focused on specific countries or continents, this systematic review provided a global perspective. Types of health professionals were not limited to a specific medical discipline. The synthesis of findings has been reported narratively, as we included different study types.

### Conclusions and Recommendations

The majority of studies included in this systematic review considered the XR-based intervention as at least noninferior to the traditional teaching methods. Most XR-based HMDs have been reported as an engaging and enjoyable tool for learners to improve their knowledge and skills. We approached this systematic review from a global perspective; however, we only found 1 study from a low-resource context using XR-based HMDs. Probably, this is an indicator that HMDs are not yet widely used in low-resource contexts, although this review shows that HMDs could provide a high quality element for medical education in LMICs. One positive aspect is that decreased access to cadavers cause high costs, which may be reduced by using XR-based HMDs for medical education [51]. Furthermore, HMDs offer the possibility of scalability and repeated practice, such as for anatomy, without adverse effects on the patient in various medical disciplines, especially in the field of surgery. The use of this technology may support the understanding of complex 3D structures, and the technology is a general training tool to prepare for the increase of technologies in the medical workspace. In addition, other disciplines such as pathology, ophthalmology, emergency medicine, gynecology, and dentistry reported effective outcomes with XR-based HMDs. XR-based HMDs can be seen as a valuable resource that has potential to strengthen medical education.

However, the deployment of HMDs in the medical setting requires further evaluation and technical advancement, for example, for HMD-based training tools. XR-based technology is still a rather novel technology, slowly unfolding its usefulness for medical education. Surgery and anatomy are quite prominent in HMD use, but it is unclear whether and how other medical disciplines may benefit, such as pediatrics. Currently, a framework or guideline for XR-based HMD interventions is lacking to guide implementations and evaluations, although initiatives like the VR-CORE international working group are working to close this gap. Further research is also needed for other requirements like financial aspects, implementation and training, technical feasibility, and reliability. Overall, based on the results of this review, XR-based HMDs seem to be a valuable component for medical education and can be recommended as an additional tool for teaching and learning particularly complex spatial structures. Based on our experience in low-income contexts, we also identified potential for the application of XR-based HMDs for medical education in LMICs, although more use cases and more research is needed.

## Appendix

Multimedia Appendix 1 PRISMA (Preferred Reporting Items for Systematic Reviews and Meta-Analyses) 2020 Checklist.

Multimedia Appendix 2 Search strings.

Multimedia Appendix 3 Data extraction.

Multimedia Appendix 4 Quality assessment.

Multimedia Appendix 5 Three levels of knowledge.

Multimedia Appendix 6 Details of number and type of study participants.

Multimedia Appendix 7 Reported effectiveness.

Multimedia Appendix 8 Benefits, shortcomings, and recommendations described within included studies.

## Abbreviations

ACROBAT-NRSI: A Cochrane Risk of Bias Assessment Tool for Non-Randomized Studies
AR: augmented reality
HMD: head-mounted device
LMIC: low- and middle-income country
MERSQI: Medical Education Research Study Quality Instrument
MR: mixed reality
NOS-E: Newcastle-Ottawa Scale-Education
PICOS: population, intervention, comparison, outcome, study design
PRISMA: Preferred Reporting Items for Systematic Reviews and Meta-Analysis
SDG: Sustainable Development Goal
SWiM: Synthesis Without Meta-analysis
VR: virtual reality
VR-CORE: Virtual Reality Clinical Outcomes Research Experts
XR: extended reality

## References

[1] World Health Organization (2019). WHO Guideline Recommendations on Digital Interventions for Health System Strengthening. WHO Guidelines Sexual and Reproductive Health.

[2] Barré J, Michelet D, Truchot J, Jolivet E, Recanzone T, Stiti S, Tesnière A, Pourcher G (2019). Virtual reality single-port sleeve gastrectomy training decreases physical and mental workload in novice surgeons: an exploratory study. Obes Surg.

[3] Barteit S, Jahn A, Banda SS, Bärnighausen Till, Bowa A, Chileshe G, Guzek D, Jorge MM, Lüders S, Malunga G, Neuhann F (2019). E-learning for medical education in Sub-Saharan Africa and low-resource settings: viewpoint. J Med Internet Res.

[4] Goh PS (2016). eLearning or technology enhanced learning in medical education-hope, not hype. Med Teach.

[5] (2018). Classification of digital health interventions v1.0 - A shared language to describe the uses of digital technology for health. World Health Organization.

[6] Frenk J, Chen L, Bhutta ZA, Cohen J, Crisp N, Evans T, Fineberg H, Garcia P, Ke Y, Kelley P, Kistnasamy B, Meleis A, Naylor D, Pablos-Mendez A, Reddy S, Scrimshaw S, Sepulveda J, Serwadda D, Zurayk H (2010). Health professionals for a new century: transforming education to strengthen health systems in an interdependent world. Lancet.

[7] Moro C, Štromberga Z, Raikos A, Stirling A (2017). The effectiveness of virtual and augmented reality in health sciences and medical anatomy. Anat Sci Educ.

[8] Pantelidis P, Chorti A, Papagiouvanni I, Paparoidamis G, Drosos C, Panagiotakopoulos T, Lales G, Sideris M (2018). Virtual and Augmented Reality in Medical Education, Medical and Surgical Education - Past, Present and Future.

[9] Jensen L, Konradsen F (2017). A review of the use of virtual reality head-mounted displays in education and training. Educ Inf Technol.

[10] (2017). What needs to be done to solve the shortage of health workers in the African Region. WHO: Regional Office for Africa.

[11] Moro C, Birt J, Stromberga Z, Phelps C, Clark J, Glasziou P, Scott A (2021). Virtual and augmented reality enhancements to medical and science student physiology and anatomy test performance: a systematic review and meta-analysis. Anat Sci Educ.

[12] Moro C, Phelps C, Redmond P, Stromberga Z (2020). HoloLens and mobile augmented reality in medical and health science education: a randomised controlled trial. Br J Educ Technol.

[13] Anyangwe S, Mtonga C (2007). Inequities in the global health workforce: the greatest impediment to health in sub-Saharan Africa. Int J Environ Res Public Health.

[14] (2008). Scaling up, saving lives - task force for scaling up education and training for health workers. World Health Organization.

[15] Asi YM, Williams C (2018). The role of digital health in making progress toward Sustainable Development Goal (SDG) 3 in conflict-affected populations. Int J Med Inform.

[16] Samadbeik M, Yaaghobi D, Bastani P, Abhari S, Rezaee R, Garavand A (2018). The applications of virtual reality technology in medical groups teaching. J Adv Med Educ Prof.

[17] Barsom EZ, Graafland M, Schijven MP (2016). Systematic review on the effectiveness of augmented reality applications in medical training. Surg Endosc.

[18] da Silva RL, Stone E, Lobaton E (2019). A feasibility study of a wearable real-time notification system for self-awareness of body-rocking behavior.

[19] Kamphuis C, Barsom E, Schijven M, Christoph N (2014). Augmented reality in medical education?. Perspect Med Educ.

[20] Cipresso P, Giglioli IAC, Raya MA, Riva G (2018). The past, present, and future of virtual and augmented reality research: a network and cluster analysis of the literature. Front Psychol.

[21] Higgins J, Thomas J, Chandler J, Cumpston M, Li T, Page M, Welch V (2019). Cochrane Handbook for Systematic Reviews of Interventions.

[22] Moher D, Liberati A, Tetzlaff J, Altman DG, PRISMA Group (2009). Preferred reporting items for systematic reviews and meta-analyses: the PRISMA statement. PLoS Med.

[23] Haddaway NR, Collins AM, Coughlin D, Kirk S (2015). The role of Google Scholar in evidence reviews and its applicability to grey literature searching. PLoS One.

[24] Facebook to acquire Oculus. About Facebook.

[25] Methley AM, Campbell S, Chew-Graham C, McNally R, Cheraghi-Sohi S (2014). PICO, PICOS and SPIDER: a comparison study of specificity and sensitivity in three search tools for qualitative systematic reviews. BMC Health Serv Res.

[26] Sterne JAC, Egger M, Smith GD (2001). Systematic reviews in health care: investigating and dealing with publication and other biases in meta-analysis. BMJ.

[27] Cochrane Library (2015). ACROBAT-NRSi (A Cochrane Risk Of Bias Assessment Tool: for Non-Randomized Studies of Interventions) for non-clinical community based studies: a participatory workshop using a worked example from public health. https://tinyurl.com/uf4jny2r.

[28] Cook DA, Reed DA (2015). Appraising the quality of medical education research methods: the Medical Education Research Study Quality Instrument and the Newcastle-Ottawa Scale-Education. Acad Med.

[29] Campbell M, McKenzie JE, Sowden A, Katikireddi SV, Brennan SE, Ellis S, Hartmann-Boyce J, Ryan R, Shepperd S, Thomas J, Welch V, Thomson H (2020). Synthesis without meta-analysis (SWiM) in systematic reviews: reporting guideline. BMJ.

[30] Wu TS, Dameff CJ, Tully JL (2014). Ultrasound-guided central venous access using Google Glass. J Emerg Med.

[31] Rai AS, Rai AS, Mavrikakis E, Lam WC (2017). Teaching binocular indirect ophthalmoscopy to novice residents using an augmented reality simulator. Can J Ophthalmol.

[32] Pulijala Y, Ma M, Pears M, Peebles D, Ayoub A (2018). Effectiveness of immersive virtual reality in surgical training-a randomized control trial. J Oral Maxillofac Surg.

[33] Peden RG, Mercer R, Tatham AJ (2016). The use of head-mounted display eyeglasses for teaching surgical skills: a prospective randomised study. Int J Surg.

[34] Luursema J, Vorstenbosch M, Kooloos J (2017). Stereopsis, visuospatial ability, and virtual reality in anatomy learning. Anat Res Int.

[35] Logishetty K, Western L, Morgan R, Iranpour F, Cobb JP, Auvinet E (2019). Can an augmented reality headset improve accuracy of acetabular cup orientation in simulated THA? A randomized trial. Clin Orthop Relat Res.

[36] Leitritz M, Ziemssen F, Suesskind D, Partsch M, Voykov B, Bartz-Schmidt K, Szurman G (2014). Critical evaluation of the usability of augmented reality ophthalmoscopy for the training of inexperienced examiners. Retina.

[37] Hooper J, Tsiridis E, Feng JE, Schwarzkopf R, Waren D, Long WJ, Poultsides L, Macaulay W, NYU Virtual Reality Consortium (2019). Virtual reality simulation facilitates resident training in total hip arthroplasty: a randomized controlled trial. J Arthroplasty.

[38] Ferrandini Price M, Escribano Tortosa D, Nieto Fernandez-Pacheco A, Perez Alonso N, Cerón Madrigal JJ, Melendreras-Ruiz R, García-Collado ÁJ, Pardo Rios M, Juguera Rodriguez L (2018). Comparative study of a simulated incident with multiple victims and immersive virtual reality. Nurse Educ Today.

[39] Ekstrand C, Jamal A, Nguyen R, Kudryk A, Mann J, Mendez I (2018). Immersive and interactive virtual reality to improve learning and retention of neuroanatomy in medical students: a randomized controlled study. CMAJ Open.

[40] Butt AL, Kardong-Edgren S, Ellertson A (2018). Using game-based virtual reality with haptics for skill acquisition. Clin Simulation Nurs.

[41] Azimi E, Winkler A, Tucker E, Qian L, Doswell J, Navab N, Kazanzides P (2018). Can mixed-reality improve the training of medical procedures?.

[42] Górski F, Buń P, Wichniarek R, Zawadzki P, Hamrol A (2017). Effective design of educational virtual reality applications for medicine using knowledge-engineering techniques. Eurasia J Math Sci Tech Ed.

[43] Bairamian D, Liu S, Eftekhar B (2019). Virtual reality angiogram vs 3-dimensional printed angiogram as an educational tool-a comparative study. Neurosurgery.

[44] Huber T, Paschold M, Hansen C, Wunderling T, Lang H, Kneist W (2017). New dimensions in surgical training: immersive virtual reality laparoscopic simulation exhilarates surgical staff. Surg Endosc.

[45] Lin Y, Yau H, Wang I, Zheng C, Chung K (2015). A novel dental implant guided surgery based on integration of surgical template and augmented reality. Clin Implant Dent Relat Res.

[46] Qin Z, Tai Y, Xia C, Peng J, Huang X, Chen Z, Li Q, Shi J (2019). Towards virtual VATS, face, and construct evaluation for peg transfer training of box, VR, AR, and MR trainer. J Healthc Eng.

[47] Yoganathan S, Finch D, Parkin E, Pollard J (2018). 360° virtual reality video for the acquisition of knot tying skills: a randomised controlled trial. Int J Surg.

[48] Huang CY, Thomas JB, Alismail A, Cohen A, Almutairi W, Daher NS, Terry MH, Tan LD (2018). The use of augmented reality glasses in central line simulation: "see one, simulate many, do one competently, and teach everyone". Adv Med Educ Pract.

[49] Rochlen LR, Levine R, Tait AR (2017). First-person point-of-view-augmented reality for central line insertion training: a usability and feasibility study. Simul Healthc.

[50] Moro C, Štromberga Z, Stirling A (2017). Virtualisation devices for student learning: comparison between desktop-based (Oculus Rift) and mobile-based (Gear VR) virtual reality in medical and health science education. Australas J Educ Technol.

[51] Stepan K, Zeiger J, Hanchuk S, Del Signore A, Shrivastava R, Govindaraj S, Iloreta A (2017). Immersive virtual reality as a teaching tool for neuroanatomy. Int Forum Allergy Rhinol.

[52] Siff L, Mehta N (2018). An interactive holographic curriculum for urogynecologic surgery. Obstet Gynecol.

[53] Bing Eric G, Parham Groesbeck P, Cuevas A, Fisher B, Skinner J, Mwanahamuntu M, Sullivan R (2019). Using low-cost virtual reality simulation to build surgical capacity for cervical cancer treatment. J Glob Oncol.

[54] Farahani N, Post R, Duboy J, Ahmed I, Kolowitz B, Krinchai T, Monaco S, Fine J, Hartman D, Pantanowitz L (2016). Exploring virtual reality technology and the Oculus Rift for the examination of digital pathology slides. J Pathol Inform.

[55] Dyer E, Swartzlander BJ, Gugliucci MR (2018). Using virtual reality in medical education to teach empathy. J Med Libr Assoc.

[56] Harrington CM, Kavanagh DO, Wright Ballester G, Wright Ballester A, Dicker P, Traynor O, Hill A, Tierney S (2018). 360° operative videos: a randomised cross-over study evaluating attentiveness and information retention. J Surg Educ.

[57] Loorbach N, Peters O, Karreman J, Steehouder M (2014). Validation of the instructional Materials Motivation Survey (IMMS) in a self-directed instructional setting aimed at working with technology. Br J Educ Technol.

[58] (2016). A practical guide to conducting research and assessment. World Health Organization.

[59] Munafo J, Diedrick M, Stoffregen TA (2017). The virtual reality head-mounted display Oculus Rift induces motion sickness and is sexist in its effects. Exp Brain Res.

[60] Albino JE, Young SK, Neumann LM, Kramer GA, Andrieu SC, Henson L, Horn B, Hendricson WD (2008). Assessing dental students' competence: best practice recommendations in the performance assessment literature and investigation of current practices in predoctoral dental education. J Dent Educ.

[61] Meng X, Yang L, Sun H, Du X, Yang B, Guo H (2019). Using a novel student-centered teaching method to improve pharmacy student learning. Am J Pharm Educ.

[62] Satava RM (2008). Historical review of surgical simulation--a personal perspective. World J Surg.

[63] Lee C, Afshari N (2017). The global state of cataract blindness. Curr Opin Ophthalmol.

[64] Huber J Virtual reality helps train emergency physicians. Scope Stanford Medicine.

[65] Kamel-ElSayed S, Loftus S (2018). Using and combining learning theories in medical education. Med Sci Educ.

[66] Monsky W, James R, Seslar S (2019). Virtual and augmented reality applications in medicine and surgery-the fantastic voyage is here. Anat Physiol Curr Res.

[67] Snoswell A, Snoswell C (2019). Immersive virtual reality in health care: systematic review of technology and disease states. JMIR Biomed Eng.

[68] von Gunten CF, Mullan P, Nelesen R, Garman K, McNeal H, Savoia M, Muchmore E, Ikeda T, Amundson S, McKennett M, Diamant J, Pepper P, Gray C, Weissman D (2017). Primary care residents improve knowledge, skills, attitudes, and practice after a clinical curriculum with a hospice. Am J Hosp Palliat Care.

[69] Fertleman C, Aubugeau-Williams P, Sher C, Lim A, Lumley S, Delacroix S, Pan X (2018). A discussion of virtual reality as a new tool for training healthcare professionals. Front Public Health.

[70] Bragard I, Guillaume M, Ghuysen A, Servotte J, Ortiz I, Pétré B (2018). [A virtual patient to improve doctor-patient communication : reality or fiction ?]. Rev Med Liege.

[71] Kisekka V, Giboney J (2018). The effectiveness of health care information technologies: evaluation of trust, security beliefs, and privacy as determinants of health care outcomes. J Med Internet Res.

[72] Murray E, Hekler E, Andersson G, Collins L, Doherty A, Hollis C, Rivera D, West R, Wyatt J (2016). Evaluating digital health interventions: key questions and approaches. Am J Prev Med.

[73] Attwell G (2006). Evaluating E-learning A Guide to the Evaluation of E-learning. Evaluate Europe Handbook Series Volume 2.

[74] Birckhead B, Khalil C, Liu X, Conovitz S, Rizzo A, Danovitch I, Bullock K, Spiegel B (2019). Recommendations for methodology of virtual reality clinical trials in health care by an international working group: iterative study. JMIR Ment Health.

