# Supplementary material for: Augmented, Mixed, and Virtual Reality-Based Head-Mounted Devices for Medical Education: Systematic Review
Source: JMIR Serious Games. 2021 Jul 8;9(3):e29080. doi: 10.2196/29080 (PMC8299342; doi:10.2196/29080)
Supplement: Multimedia Appendix 3 [file games_v9i3e29080_app3.docx]

**Multimedia Appendix 3. Extraction overview of all included studies.**

| **Title** | **Author / Year /Setting**  **/Journal** | **Study Design / Objective** | **Type of Participants/HMD**  **/Knowledge-Skills Outcome** | **Evaluation Methods / Types of Outcomes** | **Effectiveness: Medical Education** | **MERSQI NOS/**  **Risk of Bias** |
| --- | --- | --- | --- | --- | --- | --- |
| **1**  **Can Mixed- Reality Improve the Training of Medical Procedures?** | Ehsan Azimi et al. 2018  Department of Computer Science, Johns Hopkins University, Baltimore, MD, 21218, USA  40th Annual International Conference of the IEEE Engineering in Medicine and Biology Society (EMBC) | Study Design:  Controlled multi-user study  Objective:  Evaluate the advantages of using an HMD for practice of medical staff in an emergency medical area  Length of intervention: 3 weeks | Type of participants: First study: nursing students  Second study:  20 novice participants  HMD:  Microsoft HoloLens Moverio BT 300  Knowledge-Skills:  -Tourniquet application  -Needle chest decompression  -Initiate direct intravenous line | Evaluation Methods:  -2 repetitions of practical tasks (follow-up 3 weeks later)  -1st repetition: 3 questionnaires about training experience  -write down procedure steps before 2nd repetition starts  -time recording of practical steps (step correctly done, partially done, not done)  Types of Outcome:  more engagement, better user’s confidence, longer training times, better retention, no time gain | -Valuable experience for participants  -spend more time exercising  -Enables full remembrance of task steps weeks later  -However: Statistically no remarkable gap between the time needed to finalize the evaluation task which came after the training for both groups  **Effectiveness: Yes**  Mixed reality improves training | MERSQI: 12 |
| **2**  **Virtual Reality Angiogram vs3- Dimensional Printed Angiogram as an Educational tool—A Comparative Study** | David Bairamian et al. 2019  Department of Neurosurgery, Nepean Hospital, The University of Sydney, Sydney, Australia  Neurosurgery, Volume 85, Issue 2, August  2019, | Study Design Comparative Study  Objective:  Analyze and compare the usability and the efficiency of 3D printed models and VR models in a neurosurgical training environment  Length of intervention: 1-2 days | Type of participants  -10 neurosurgical trainees  -1 qualified neurosurgeon  HMD:  -Google Daydream,  -app programming in C#  -Unity platform 5.5.0  Knowledge /Skills: Knowledge Acquisition: Aneurysms in Neurosurgery | Evaluation Methods Two studies:  -*Validation of models:* Tester asked questions (90s per answer) -time recording – followed by Likert-like questionnaire  - *Measure learning curve effect* – *VR angiogram*: VR naïve, practicing neurosurgeon tests 15 models  -test time recorded  Statistics: T-test, paired t-test, Pearson correlation coefficient, regression analysis, cumulative sum test  Types of outcome:  -VR angiogram: better resolution, ability to zoom  -3D printed model: better depth perception, manipulation | -VR angiogram  In specific cases a stereoscopic depth perception may be useful in anatomy training. A viable solution could be a VR angiogram instead of printed 3D models which are used for training and for preoperative planning.  -Implementation Easy to implement  -Resolution and Zoom Outstanding resolution also has a zoom  **Effectiveness: Yes, in selected cases** | MERSQI: 13  NOS: 5  Risk of Bias: Low |
| **3**  **Virtual Reality Single-Port Sleeve Gastrectomy Training Decreases Physical and Mental Work load in Novice Surgeons: An Exploratory Study** | Jessy Barré et al. 2019  Ilumens, Université Paris Descartes Paris France  et al.  Springer Nature | Study Design Exploratory Study  Monocentric-controlled trial  Objective:  -Evaluate the effect of the VR training tool on “physical and mental workload” (p.1309) on inexperienced surgeons  -VR simulator evaluation  Length of intervention January2018  to September2018 | Participants:  Ten residents in surgery (same level of experience)  HMD:  HTC Vive  Knowledge /Skills: Perform a Single-Port Sleeve Gastrectomy | Evaluation Methods  -two groups, VR and control group  -participation in real case of SPSG,  -second case 1 month later.  -VR group: training between two surgeries  - Self-assessment questionnaires about mental and physical loads: NASA-TLX, Borg scale, and manikin discomfort test  -Evaluation VR simulator: presence, cybersickness, and usability questionnaires Statistics:  Mann-Whitney U-test, Friedman test,  Wilcoxon Test Types of Outcome:  -decrease of mental demand between 1st and 2nd surgery  -VR simulator: realistic, useful | Objective 1:  -Study revealed a decrease of the mental requirements and dimensions of effort in the VR group between the first and the second surgery  -During the second surgery, a slightly significant difference could be observed referring to the mental demand between the two groups  - Postural discomfort of the VR group became less with practice (P < .01), mainly between the first and the second surgery (P < .05).  The VR simulator  The team members identified the VR simulator as practical, usable, and very helpful to learn surgery  **Effectiveness: Yes** | MERSQI: 11.5 |
| **4**  **Using Low-Cost Virtual Reality Simulation to Build Surgical Capacity for Cervical Cancer Treatment** | E. Bing et al.  2019  Zambia  *Journal of Global Oncology* | Study Design Iterative user-centered Design  Objective  Evaluate if low-cost VR simulators can be useful to train surgeons in low- resource environments carrying out cancer surgery  Length of intervention: 2-3 weeks  preparation 9 months (part time) | Type of participants  -10 novice surgeons, senior medical students and resident trainees in obstetrics and gynecology and fellow in gynecologic oncology, United States and Zambia  HMD  VR simulation with Oculus Rift and hand controllers  Knowledge /Skills Outcome:  Perform virtual radical abdominal hysterectomy | Evaluation Methods  -Five steps of a virtual radical abdominal hysterectomy  -Summary screen at the end of simulation: Measurement of time and movement efficiency  -written assessment after each session to identify areas of improvement  Types of Outcome:  Better time and movement efficiency | Virtual hysterectomy successfully performed  Remarkable improvement in executing a virtual hysterectomy by surgical trainees in Zambia: enhancement in time effectiveness and notable movement  **Effectiveness: Yes**  Low-cost VR could be a very useful instrument for surgical novices to better understand complicated surgical oncology methods. For global surgical cancer treatment low-cost VR could be an answer. | MERSQI: 10  NOS: 5  Risk of Bias: Low |
| **5**  **Using Game- Based Virtual Reality with Haptics for Skill Acquisition** | Ann L. Butt et al. 2018  University of Utah College of Nursing, Salt Lake City  *Clinical Simulation in Nursing,* [Volume 16,](https://www.sciencedirect.com/science/journal/18761399/16/supp/C) March 2018,Pages 25-  32  International Nursing Association for Clinical Simulation and Learning. Published by Elsevier Inc. | Study Design  Mixed methods pilot study using a triangulation design convergence model Objective:  Investigate the usability of a game-based VR system intended to exercise urinary catheterization and examine the reaction of the users  Length of intervention 2 weeks and more… | Participants 20 junior-level  undergraduate nursing students in the 5th or 6th semester  Second group: students (2 institutions)  HMD  Samsung Gear (and interactive gloves)  Knowledge /Skills Outcome: Perform urinary catheterization | Evaluation Methods  2 groups - initial survey used to assign 10 participants to each group  -control group: 1-hour practice - feedback  -VR group: 1-hour practice - feedback  -Survey user experience and system usability  - survey data combined with observation for interpretation  -2 weeks later: practical recorded session- scored by blinded reviewer using checklist  System Usability Survey (SUS) Faculty-designed user-reaction survey  Types of Outcome:  Ease of use, fun, game better than task trainer, lose track of time, l, focus during practice, willingness to practice repeatedly, VR group completed more procedures in 1 hour and spent more time practicing | Students who took part in the study were convinced by piloted VR system and their usability ratings showed their readiness and commitment to make use of the VR tool for skill practice  Development of procedural skills is useful for students to repeatedly exercise procedural skills and as a result improve their skill retention  **Effectiveness: Yes** | MERSQI: 14 |
| **6**  **Using virtual reality in medical education to teach empathy** | Elizabeth Dyer et al. 2018  University of New England  *Journal of the Medical Library Association* | Study Design Educational project  Objective:  Approve technology to train medical students to be understanding to older adults, using VR software that allows them to dissemble a patient with age-related diseases  Length of intervention: More than 2 years | Participants:  Student library workers and volunteer medical students. First-year: 178 first-year medical students  -second project year: inclusion of first-year physician assistant students HMD  Oculus Rift  Knowledge /Skills Outcome:  Develop empathy and understanding for older people | Evaluation Methods  -Pre- and post-assessments in REDCap  -Own assessment developed  Types of Outcome:  Better understand age-related health problems | New VR teaching approach  The project successfully established a new teaching approach to the medical curricula. The outcome was that VR improves the students’ awareness of age-related health issues and raised their understanding for older people suffering from loss of vision and loss of hearing or Alzheimer’s.  **Effectiveness: Yes**  VR teaching method helps medical and health professions students understand health problems and develop empathy for others | MERSQI: 7.5  NOS: 1  Risk of Bias: Serious |
| **7**  **Immersive and interactive virtual reality to improve learning and**  **retention of neuroanatomy in medical students: a randomized controlled study** | Chelsea Ekstrand et al.  University of Saskatchewan, Canada  *CMAJ Open, 2018* | Study Design:  Randomized controlled study  Objective:  Analyze the influence of immersive – VR to improve  learning of neuroanatomy and compare the results to conventional paper-based methods.  Length of intervention: 14 days | Participants 64 Participants  first- or second-year medical students  HMD:  HTC Vive  Knowledge /Skills Outcome:  Understand brain structures (neuroanatomy) | Evaluation Methods  - two groups – VR: 31, Paper- based:33  -baseline test  -VR training followed by VR learning - neuroanatomy  - Paper-based learning  -Postintervention test  -Satisfaction survey  -Postintervention test (5-9 days later)  Types of Outcome:  No significant differences between groups  Neurophobia decreased | Neuroanatomy training   - can contribute to improving knowledge achievement and retention - may enhance motivation to learn - may decrease neurophobia   -No remarkable differences between the two groups. Results from satisfaction survey made obvious that neurophobia seemed to have decreased.  **Effectiveness**  **VR tool may be used additionally** | MERSQI: 13.5 |
| **8**  **Exploring virtual reality technology and the Oculus Rift for the examination of digital pathology slide** | N. Farahani et al.  2016  University of Pittsburgh, USA  J Pathol Inform 2016, 1:22 | Study design:  Proof of concept and feasibility study  Objective:  Investigate the use of Oculus Rift for analyzing pathology slides in a VR environment  Length of intervention: 2 weeks | Participants  3 pathologists  HMD  Oculus Rift DK2,  Glass slides from twenty lymph node cases (ten with benign and ten malignant diagNOSes) digitized using a WSI scanner  Knowledge /Skills Outcome:  Ability to examine pathology slides | Evaluation Methods  -Pathologists read slides in both modalities  -diagNOStic concordance and time to read measured (stopwatch)  -data recorded in Google forms sheet  -pathologists rated ease of navigation, diagNOStic confidence, image quality (scale 1-10)  - data collection on a slide/case basis -extraction of overall values Statistics: unpaired t-test  Types of Outcome:  -90% diagNOStic concordance  -Ratings for image quality and diagNOStic confidence higher for traditional system | Oculus Rift DK2  -can be used in a virtual environment for viewing and navigating pathology slide images  -feasible for diagNOStic reasons however:  -limited image resolution  -navigation difficulties  -viewing limitations using mobile computers  **Effectiveness: Feasible, with restrictions** | MERSQI: 8.5  NOS: 3  Risk of Bias: Serious |
| **9**  **Comparative study of a simulated incident with multiple victims and immersive virtual reality** | Ferrandini Price Mariana et al.  2018  Murcia, Spain  *Nurse Education Today* | Study Design Comparative study  Objective:  -Validate the efficiency in the execution of the START triage and compare VR to Clinical Simulation in a Mass Casualty Incident  -Determine the pressure put on health professional s resulting from the stressful situations described  Length of intervention: 1 year | Participants  67 students and health professionals. Average length professional experience 13 years, of which 10 in emergency care  HMD  Samsung Gear VR, Samsung Galaxy S6 Knowledge /Skills Outcome:  Open an airway/hemorrhage compression | Evaluation Method  -Same simulation cases – 1st group and 2nd (VR) group  -Performance of life saving maneuvers (open an airway/hemorrhage compression)  -Tracking of action scenes with camera  -salivary amylase test before and after the triage  Statistics:  Wilcoxon (W) signed-rank test, Student’s t-test, Chi-squared test Types of Outcome:  -both groups successful in the triage with minimal differences  -amylase test clarifies that clinical simulation is stressful. | -VR used for training   - pedagogic resource - training of health professionals   -Clinical simulation   - generates a demanding training experience - should not be replaced by virtual reality   **Effectiveness**   - **unclear** - **VR as additional training tool** | MERSQI: 13  NOS: 6  Risk of Bias: Low |
| **10**  **360 Operative Videos: A Randomized Cross-Over Study Evaluating Attentiveness and Information Retention** | Cuan M. Harrington et al.  2018  Royal College of Surgeons in Ireland  *Journal of Surgical Education* | Study Design Randomized Cross-Over Study  Objective  Evaluate the difference in attentiveness between 360º and 2D video formats  Length of intervention: June to July 2017 | Participants  Preclinical undergraduate students from a medical university in Ireland.  HMD  Samsung Gear VR  2D experience on a 75- inch television  Knowledge /Skills Outcome:  Acquisition of knowledge: laparoscopic cholecystectomy | Evaluation Methods  -360-degree video shows laparoscopic cholecystectomy, comparison with 2D format  -group A Samsung Gear VR, group B television  -both groups watched both videos (cross-over) -completion of questionnaires for feedback on experiences  Types of Outcome:  Higher engagement, attentiveness Entertainment minimize task unrelated thoughts | Attentiveness associated with 360º and 2D video formats   - higher attentiveness with 360° format - overall positive feedback - 360° experience was entertaining - 360° format beneficial to learning.   **Effectiveness: Yes**  **- but: no big differences in information retention** | June to July 2017  MERSQI: 10.5 |
| **11**  **Virtual Reality Simulation Facilitates Resident Training in Total Hip Arthroplasty: A Randomized Controlled Trial** | Jessica Hooper et al. 2019  New York University Langone Health, New York  *Journal of Arthroplasty* | Study Design  Randomized Controlled Trial  Objective  Compare the enhancement in cadaver THA performance, clearly defined aspects of surgical skills, knowledge and understanding of surgical anatomy and evidences  Length of intervention: 4-5 weeks | Participants Fourteen PGY-1 orthopedic residents  HMD  Oculus Rift CV1 with hand controllers  Knowledge /Skills Outcome  Perform Total Hip Arthroplasty | Evaluation Methods  --REDCap pretest  -1st and 2nd cadaver sessions  -REDCap posttest  -Surgical competency measured by checklist adapted from different general arthroplasty checklists.  -4 arthroplasty surgeons evaluated checklists.  Statistics:  Statistical evaluation with T-test and Mann-Whitney U tests Types of Outcome:  -Significant improvement in technical skills for PGY-1 residents  -medical knowledge not improved | VR simulation   - enhances surgical skills - has no remarkable effect on medical knowledge   **Effectiveness:**  **Yes – concerning technical skills Unclear – no remarkable influence on medical knowledge** | MERSQI: 12 |
| **12**  **The use of augmented reality glasses in central line simulation: “see one, simulate many, do one competently, and teach everyone”** | C. Huang et al.  2018  Tertiary-care urban teaching hospital, Looma Linda, USA  *Advances in Medical Education and Practice, Dovepress* | Study Design Prospective randomized controlled study  Objective:  Evaluate the practicability of using augmented reality (AR) glasses in central line simulation by novice operators. Compare its efficacy to standard central line simulation/ teaching. | Participants  32 adult novice central line operators, medical doctors, respiratory therapists, and sleep technicians recruited from the medical field HMD  Brother AirScouter Knowledge /Skills Outcome  Placement of central venous catheter | Evaluation Methods  -Pre-questionnaire  -Watch central line instructional video, -Control group: Central line simulation, -AR group: AR video and tutorial  -central line simulation, questionnaire  -observer recorded compliance with checklist  Statistics: Kolmogorov-Smirnov test, Pearson’s Chi square test, Mann-Whitney test  Types of Outcome:  -easy to use when placing central lines, decrease procedure time, decrease number of attempts, helpful in medical curricula  -easy to use when placing central lines, decrease procedure time, decrease number of attempts, helpful in medical curricula | AR simulation of central line catheters in dummies as an educational tool is feasible and effective in novice operators. Adherence level in the AR group vs non-AR group   - notable difference - remarkable increase is because the AR group received feedback as they execute the procedure   **Effectiveness: Yes** | MERSQI: 11.5 |
| **13**  **New dimensions in surgical training: immersive virtual reality laparoscopic simulation exhilarates surgical staff** | Tobias Huber et al. 2017  Universität Mainz  Online Springer Science+Business Media New York 2017 | Study Design Clinical and technical feasibility study  Objective  Establish a new combined highly IVR laparoscopy setup  Investigate first experiences with reference to the degree of involvement, motion sickness, and performance measurements. | Participants  10 members of surgical department  HMD  HTC Vive, Knowledge /Skills Outcome  Perform fine dissection, peg transfer, cholecystectomy | Evaluation Methods  -perform 3 tasks (fine dissection, peg transfer, cholecystectomy) on VR simulator  -task repeated in VR environment  - questionnaires about immersion and motion sickness  Types of Outcome:  Participants time longer during VR session, cholecystectomy higher error rates, no motion sickness, high level of exhilaration, no distraction, complete focus, presence: | VR laparoscopic simulation: Participants were enthusiastic about:   - high level of immersion - clinical feasibility   Setup of laparoscopic simulation enables totally new surgical training.  **Effectiveness: Yes**,   - further investigations required | MERSQI: 10.5  NOS: 4  Risk of Bias: Low |
| **14**  **Critical Evaluation of the Usability of Augmented Reality Ophthalmoscopy for the training of inexperienced examiners** | Martin A. Leitritz et al. 2014  Centre for Ophthalmology, University Eye- Hospital,  Tuebingen, Germany  Retina, the journal of retinal and vitreous diseases | Study Design Evaluation  Objective  -Analyze the usability of the Eyesi indirect system for learning and practicing the ophthalmoscopic examination for inexperienced medical students  -Introduce a method to objectively determine the diagNOStic skills after training in binocular indirect ophthalmoscopy | Participants  Thirty-seven students (fourth year of medical school), without any previous experience in binocular ophthalmoscopy HMD  ARO group: **Eyesi indirect system** Knowledge /Skills Outcome Ophthalmoscopic examination | Evaluation Methods  -two groups control group, AR group, examine a real patient   - questionnaire - students produced drawings of the disk - output criterion for the evaluation of examination skills - Semi-quantitative assessment of drawings   Statistics:  Types of Outcome:  No differences in questionnaire, objective analysis showed better values for AR group | AR training simulator   - feasibility to work out ophthalmoscopy training for students who have no experience in indirect ophthalmoscopy Better performance with a simulator - Objective evidence that students performed outstandingly better with a simulator after only one short training   **Effectiveness: Yes** | MERSQI: 13 |
| **15**  **A Novel Dental Implant Guided Surgery Based on Integration of Surgical Template and Augmented Reality** | Yen-Kun Lin, et al. 2015  National Chung Cheng  University, Chia-yi, Taiwan  *Wiley Periodicals* | Study Design In vitro study  Objective  -Establish an augmented reality-based dental implant placement system  -Evaluate the precision of the virtually planned versus the  actual prepared implant site created in vitro. | Participants  54 and 45-old patient HMD:  Sony HMZ-T1, customized surgical environment for dentistry Knowledge /Skills Outcome:  AR-based dental implant placement | Evaluation Methods  - CT generated scan images measured the deviation between planned and prepared positions  Statistics:  Shapiro-Wilk test, ANOVA Types of Outcome:  accuracy sufﬁcient for clinical practice, transfer precision of three- dimensional implant planning | Augmented reality technology helps reduce deviation of implant Integration of augmented reality technology and surgical template considerably reduced the deviation of implant placement from planned position.  Accuracy of computer-aided implant surgery increased  A combination of stereoscopic visualization together with head- mounted displays may raise the precision of computer-aided implant surgery  **Effectiveness: Yes** | MERSQI: 10  NOS: 2  Risk of Bias: No Information |
| **16**  **Can an Augmented Reality Headset Improve Accuracy of Acetabular Cup Orientation in Simulated THA? A Randomized Trial** | Kartik Logishetty et al. 2018  Orthopaedical university hospital  *Clinical Orthopaedics and Related Research* | Study Design  Randomized Simulation Trial  Objective  -Discover if an AR headset enhances the accuracy of acetabular component positioning compared with hands-on training by an expert surgeon  -Evaluate the learners’ impression on the AR headset regarding the realism of the task, its potential role for surgical training  Length of intervention: 4 weeks | Participants Twenty-four study  participants (medical students’ final year, applying to surgery residency programs, no prior arthroplasty experience)  HMD:  Microsoft HoloLens  Knowledge /Skills Outcome:  Perform acetabular cup orientation in THA | Evaluation Methods  two groups, on group AR, control group instructed by a surgeon  -4 sessions in 4 weeks, assessment, training, reassessment  - measure errors between the planned and achieved placement Statistics:  Types of Outcome:  AR group less errors, AR platform effective for development of visuospatial skills, preferred combination: experienced trainer and AR for self-teaching | AR useful in education  -Participants were trained by AR or a surgeon  -AR as a supplement to expert instructions in the operating room is regarded as a feasible and beneficial training tool  - AR could be a useful tool in education as it shows that motor skills for hip replacement may be learned in an unsupervised environment  **Effectiveness: Feasible** | MERSQI: 11.5 |
| **17**  **Stereopsis, Visuospatial Ability, and Virtual Reality in Anatomy Learning** | J. Luursema et al.  2017  Department of Anatomy, Nijmegen, Netherlands  *Anatomy Research International, Volume 2017, Article ID*  *1493135, 7 pages* | Study Design Randomized Control Trial  Objective  -Investigate to what extent stereopsis can contribute to digital anatomy learning  -Analyze the effectiveness of virtual reality for anatomical learning for users of differing visuospatial ability  Length of intervention: Few days | Participants  63 medical and biomedical students  HMD:  Oculus Rift SDK 2 | Evaluation Methods  -two groups,  -one group with HMD,  -one group without,  -posttest Statistics: SPSS; ANOVA  Types of Outcome:  -Impact of visuospatial ability on correct answers  - no impact of cognitive load on performance  Knowledge /Skills Outcome: Learning anatomy | VR for anatomy learning   - unverified if the use of VR helps or impedes anatomy learning - further research into use of VR for anatomy learning needs to be done   **Effectiveness: Not proven** | MERSQI: 11.5 |
| **18**  **Virtualisation devices for student learning:**  **Comparison between desktop-based (Oculus Rift) and mobile-**  **based (Gear VR) virtual reality in medical and health science education** | C. Moro et al.  2017  Faculty of Health Sciences and Medicine, Bond University Queenland, Australia  *Australasian Journal of Educational Technology* | Study Design Randomized Control Trial  Objective  -Compare the test results of an anatomical knowledge test between two virtual reality headsets, the Oculus Rift and Gear VR  -Analyze students understanding and bad health effects which appeared during the lesson. | Participants 20 students  HMD:  Oculus Rift / Samsung Gear  Knowledge /Skills Outcome:  Acquisition of anatomical knowledge | Evaluation Methods  -two groups, one with Oculus Rift, one with Samsung Gear   - lesson on spine anatomy - Questionnaires (one Likert scale)   Statistics:  t-test, Mann-Whitney U Test Types of Outcome:  -no significant difference between HMDs  -fuzzy vision using Samsung Gear VR | Comparison between desktop-based (Oculus Rift) and mobile-based (Gear VR)   - no important differences in test scores from students using either device   -40% of students noted considerably higher rates of blurred vision and nausea using the Gear VR   - the cost-effective Gear VR was as appropriate for teaching isolated systems as the more expensive Oculus Rift   **Effectiveness: Yes**  Samsung Gear VR is less expensive and as suitable as Oculus Rift.  Considerably higher rates of blurred vision and nausea using the Gear VR. | MERSQI: 10.5 |
| **19**  **The use of head- mounted display eyeglasses for teaching surgical skills: A prospective randomized study** | Robert G. Peden et al. 2016  Princess Alexandra Eye Pavilion and Department of Ophthalmology, University of Edinburgh  *International Journal of Surgery 34 (2016)*  *169-173* | Study design Prospective randomized study  Objective  Evaluate whether videos provided via head-mounted displays can enhance skill acquisition and satisfaction in basic surgical training compared to conventional teaching. | Participants  14 medical students with no prior suturing experience in surturing  HMD:  Google Glass  Knowledge /Skills Outcome  Improve suturing skills | Evaluation Methods  -3 groups, conventional, HMD assisted, HMD self-teaching   - instruction surturing, 15 minutes practice   -suturing recorded, graded by masked assessor, skill score (1-10)   - questionnaire: confidence and satisfaction   Statistics:  Types of Outcome: Enjoyable,  HMD self-teaching as effective as conventional teaching | Comparison HMD to conventional teaching   - acquisition of surgical ability could not be improved by use of HMD - HMD more enjoyable compared to traditional teaching - Self-learning with HMD gives equivalent skill acquisition to regular teaching   **Effectiveness: No**  VR learning is more entertaining | MERSQI: 12.5 |
| **20**  **Effectiveness of Immersive Virtual Reality in Surgical Training – A Randomized Control Trial** | Pulijala et al. 2018  UK  University of Huddersfield, United Kingdom et al.  J Oral Maxillofac Surg 76:1065-1072, 2018  Journal of Oral and Maxillofacial Surgery,  Volume 76, Issue 5,  May 2018, Pages 1065-  1072 | Study design  Randomized Control Trial  Objective  Evaluate the impact on knowledge and self- confidence of students when using VR surgery | Participants  95 residents from 7 dental schools  Study group and control group  HMD:  Oculus Rift  and Leap Motion devices  Knowledge /Skills Outcome:  Le Fort I Osteotomy | Evaluation Methods  -two groups, study group and control group  -comparative evaluation before and after the intervention  -3 questionnaires (one Likert scale)  -objective assessment of cognitive skills  Statistics: ANOVA  Types of Outcome:  -greater self-confidence  -novices greatest improvement | Influence on self-confidence  -The participants of the study group showed much higher self-confidence levels than the control group  -First year participants demonstrated the greatest improvement in their confidence compared with participants in their second and third year  **Effectiveness: Yes** | MERSQI 14.5 |
| **21**  **Towards Virtual VATS, Face, and Construct Evaluation for Peg Transfer Training of Box, VR, AR, and MR Trainer** | Zhibao Qin et al. 2019  Department of thoracic surgery of Yunnan First People’s Hospital Kunming 650000, China  *Hindawi*  *Journal of Healthcare Engineering*  *Volume 2019, Article*  *ID 6813719, 10 pages* | Objective  Evaluate differences in peg transfer training for CVR (Cognitive VR), VR, AR, MR | Participants  32 medical trainees, 24 novices and 8 experts  HMD  Peg transfer training, CVR and MR training with HMD device HTC Vive Surgical environment: Samsung Gear  Knowledge /Skills Outcome:  Peg transfer training | Evaluation Methods  -Peg transfer procedure on five simulators  -introduction experts and novices  -questionnaires (partly Likert scale) Statistics:  t-test, Shapiro-Wilk test Types of Outcome:  -AR Trainer: better haptic fidelity and accuracy  -Box trainer/MR trainer: best 3D perception and surgical immersive performance  -VR trainer: better performance than traditional trainer Knowledge /Skills Outcome: Peg transfer training | Medical simulation systems   - Medical simulation systems could contribute to a more immersive and successful training environment - It turns out that the comparison of the six parameters is not comprehensive enough because in the comparison experiments design, the box simulator is not able to automatically record the angle parameters of the instrument   **Effectiveness: Yes** | MERSQI: 12.5  NOS: 6  Risk of Bias: Low |
| **22**  **Teaching binocular indirect ophthalmoscopy to novice residents using an augmented reality simulator** | Amandeep S. Rai  Toronto Ophthalmology Residents Introductory Course (TORIC)  *CAN J OPHTHALMOL—VOL. 52, NO. 5, OCTOBER*  *2017, pages 430-434* | Study design Prospective Randomized Control Trial  Objective  Make a comparison between the conventional teaching approach (BIO) and the AR BIO Simulator | Participants:  28 post-graduate year one (PGY1) ophthalmology residents  HMD:  EyeSI BIO simulator with headpiece Knowledge /Skills Outcome:  Skill acquisition ophthalmoscopy | Evaluation Methods  -two groups  -evaluation on simulator  -3 tasks  -outcome measure: total raw score, total time elapsed, performance Types of Outcome:  AR group better scores and performance | EyeSI AR Bio simulator  - compared to conventional teaching  -the EyeSI AR Bio simulator may be superior for novice ophthalmology students  **Effectiveness: Yes** | MERSQI: 12.5 |
| **23**  **First Person Point of View Augmented Reality for Central Line Insertion Training: A Usability and Feasibility Study** | Lauryn R. Rochlen 2017  Department of Anesthesiology, University of Michigan  *Simul Healthc. Author manuscript; available in PMC 2018 February 01* | Study design  Usability and feasibility study  Objective  Assess the usability and feasibility of AR technology as a part of medical skills training on needle insertion (CVC) | Participants  Forty subjects, including medical students and anesthesiology residents HMD:  Epson Moverio BT-200 Knowledge /Skills Outcome:  Central Venous Catheter placement | Evaluation Methods  -training with AR glasses – CVC placement  -practical test without HMD  -procedure documented  -participants completed a survey Statistics: Chi-square test  Types of Outcome:  Realistic, helpful, promotion of learning, should be integrated into medical curricula | AR technology for medical skills training  AR technology is a potentially important supplement to medical skills training as the usability and feasibility results showed.  A significant next step would be the evaluation of this technology and its further development  **Effectiveness: Yes** | MERSQI: 8.5  NOS: 6  Risk of Bias: Low |
| **24**  **An Interactive Holographic Curriculum for Urogynecologic Surgery** | L.Siff and N. Mehta 2018  American College of Obstetrics & Gynecology  Obstetrics & Gynecology: October 2018 - Volume 132 - Issue - p 27S-32S | Objective  Evaluate the outcome of interactive holographic modules for urogynecologic surgery. | Participants 18 residents HMD:  MS Hololens Knowledge /Skills Outcome:  Learn surgical anatomy: ligament suspension and sacropinous ligament fixation | Evaluation Methods  -preparedness survey  -knowledge test (pre and post-test) Likert-based questionnaires  Types of Outcome:  -in comparison to traditional methods ranked “much” or “very much better”  -81% would use HMD for surgery planning | Interactive holographic curriculum   - teaches complex pelvic floor anatomy, management of potential complications of the “uterosacral ligament suspension” and (p.28)“sacrospinous ligament fixation” - widely accepted by gynecologic subspecialty colleagues Advantage of HMDs - gather and combine data such as online media, videos, textbooks, mobile applications and condense data into one single device - can save time and is cost-efficient   **Effectiveness: Yes** | MERSQI: 11  NOS: 5  Risk of Bias: Low |
| **25**  **Immersive virtual reality as a teaching tool for neuroanatomy** | K. Stephan et al 2017  Department of Otolaryngology, Icahn School of Medicine at Mount  Sinai, New York, USA  *International Forum of Allergy & Rhinology, Vol. 7, No. 10* | Study design  Randomized Control Trial Objective  Compare VR model as a teaching tool for neuroanatomy  Length of intervention: 8 weeks | Participants  66 medical students  HMD:  Oculus Rift  Knowledge /Skills Outcome: Acquisition of neuroanatomical knowledge | Evaluation Methods  -two groups  -one VR, one online textbooks  -evaluation: assessment  -pre-intervention quiz  -post-intervention quiz retention quiz, 8 weeks later  Statistics: Mann-Whitney U test Types of Outcome:  -no significant difference concerning acquisition of anatomical knowledge  -but: enjoyable, engaging, useful | Teaching tool for neuroanatomy   - regarding student exam performance there is no difference between the VR model of neuroanatomy and conventional teaching methods - various subjective measurements showed that the VR tool was superior in the following areas: engagement, enjoyment, usefulness, and learner motivation - as VR technologies are reaching a higher level of quality and are becoming more accessible, it will be more likely to integrate VR into anatomy teaching.   **Effectiveness: Not clear** | MERSQI: 11.5 |
| **26**  **Ultrasound in Emergency Medicine** | Teresa S. Wu et al. 2014  Phoenix, Arizona  *The Journal of Emergency Medicine, Vol. 47, No. 6, pp. 668–*  *675, 2014* | Study design Feasibility study  Objective  Assess if ultrasound- procedures using Google Glass would be effective in Emergency Medicine | Participants:  40 participants of varying training levels  HMD:  Google Glass Knowledge /Skills Outcome:  Perform ultrasound-guided central line | Evaluation Methods  -two groups  -1st group Google Glass to perform ultrasound-guided central line.  2nd group used traditional ultrasound during the procedure  -analysis of video recordings of eye and hand movements  Statistics:  Types of Outcome:  1st group took longer to gain access, more needle redirections, less head movements | -Ultrasound   - ultrasound-guided procedures with Google Glass can be executed - on average Google Glass wearers needed longer to gain access and they had more needle redirections, but fewer head movements - wearable technology can be integrated into clinical practice by medical trainees’ practitioners with various levels of experience and then perform ultrasound-guided procedures using Google Glass   **Effectiveness: Not clear** | MERSQI: 14.5 |
| **27**  **360° virtual reality video for the acquisition of knot tying skills: A randomised controlled trial** | S. Yoganathan et al 2018  Royal College of Surgeons, England  International Journal of Surgery 54, 24-27. | Study design  Randomized Control Trial  Objective  Compare a 360° VR video for knot tying skills to 2D video teaching | Participants 40 physicians  HMD:  Headset without branding VR video or 2D video  Knowledge/Skills Outcome  Acquisition of knot tying skills | Evaluation Methods  -2 groups  -1st group 360-degree video  -2nd group 2D video teaching  -watch video (15 min)  -tie reef knot  -assessment by blinded assessor Statistics: Mann-Whitney U Test Types of Outcome:  Application of 360-degree VR video successful  As independent teaching aid or as adjunct | Advantage of 360-degree VR video technology in surgical training   - independent teaching aid - supplement to conventional teaching - significantly better knot tying scores in the VR video teaching arm compared with conventional procedure   **Effectiveness: Yes** | MERSQI: 12.5 |
