# Supplementary material for: Augmented, Mixed, and Virtual Reality-Based Head-Mounted Devices for Medical Education: Systematic Review
Source: JMIR Serious Games. 2021 Jul 8;9(3):e29080. doi: 10.2196/29080 (PMC8299342; doi:10.2196/29080)
Supplement: Multimedia Appendix 4 [file games_v9i3e29080_app4.docx]

**Multimedia Appendix 4. Quality assessment**

**
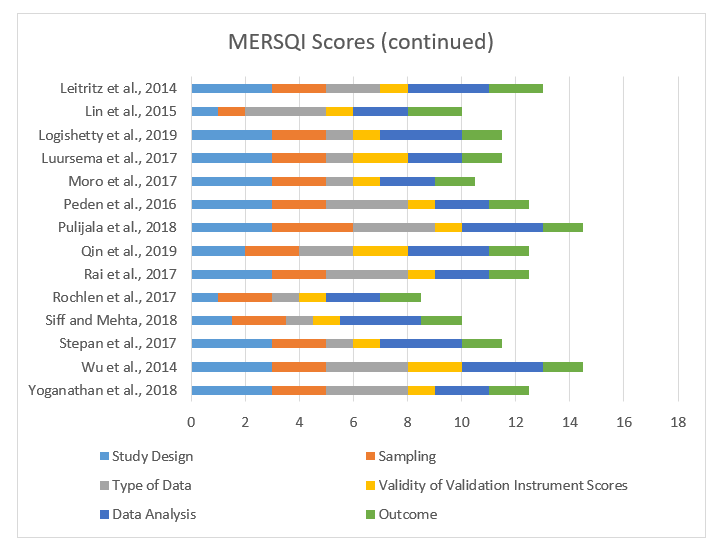
Medical Education Research Quality Instrument (MERSQI) scores**


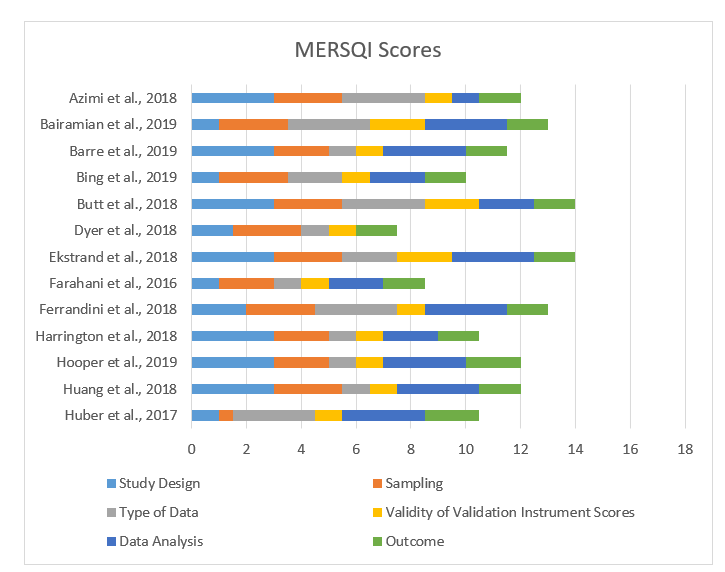


**Figure 1.** Medical Education Research Quality Instrument (MERSQI) scores, studies 1-13 with scores ranging from 5-18

**Newcastle-Ottawa Scale-Education (NOS-E) scores**

NOS-E Scores

Azimi et al., 2018

Bairamian et al., 2019 Barre et al., 2019 Bing et al., 2019 Butt et al., 2018 Dyer et al., 2018 Farahani et al., 2016

Ferrandini et al., 2018 Huber et al., 2017 Leitritz et al., 2014 Lin et al., 2015 Peden et al., 2016 Qin et al., 2019 Rochlen et al., 2017 Siff and Mehta, 2018 Wu et al., 2014

0

1

2

3

4

5

6

Representativeness

Selection of Comparison Group

Comparability of Comparison Group Blinding of Assessment

Study Retention

|  |  |  |  |  |  |
| --- | --- | --- | --- | --- | --- |
|  |  |  |  |  |  |
|  |  | |  |  |  |
|  |  |  |  |  |  |
|  |  |  |  |  |  |
|  |  |  |  |  |  |
|  |  |  |  |  |  |
|  |  |  |  |  |  |
|  |  |  |  |  |  |
|  |  |  |  |  |  |
|  |  |  |  |  |  |
|  |  |  | |  |  |
|  |  |  | |  |  |
|  |  |  |  |  |  |
|  |  |  |  |  |  |

**Figure 2.** Newcastle-Ottawa Scale-Education (NOS-E) scores of non-randomized studies with scores ranging from 0-6.

**Risk of bias assessment for non-randomized studies**

**Table 1.** Risk of bias assessment for non-randomized studies.

| **Study** | **Risk of Bias** | **Study** | **Risk of Bias** |
| --- | --- | --- | --- |
| Azimi et al., 2018 | Low | Huber et al., 2017 | Low |
| Bairamian et al., 2019 | Low | Leitritz et al., 2014 | Low |
| Barre et al., 2019 | Low | Lin et al., 2015 | No information |
| Bing et al., 2019 | Low | Peden et al., 2016 | Low |
| Butt et al., 2018 | Low | Qin et al., 2019 | Low |
| Dyer et al., 2018 | Serious | Rochlen et al., 2017 | Low |
| Farahani et al., 2016 | Serious | Siff and N. Mehta, 2018 | Low |
| Ferrandini et al., 2018 | Low | Wu et al., 2014 | Low |
