# Supplementary material for: Augmented, Mixed, and Virtual Reality-Based Head-Mounted Devices for Medical Education: Systematic Review
Source: JMIR Serious Games. 2021 Jul 8;9(3):e29080. doi: 10.2196/29080 (PMC8299342; doi:10.2196/29080)
Supplement: Multimedia Appendix 5 [file games_v9i3e29080_app5.docx]

**Multimedia Appendix 5. Three levels of knowledge.**


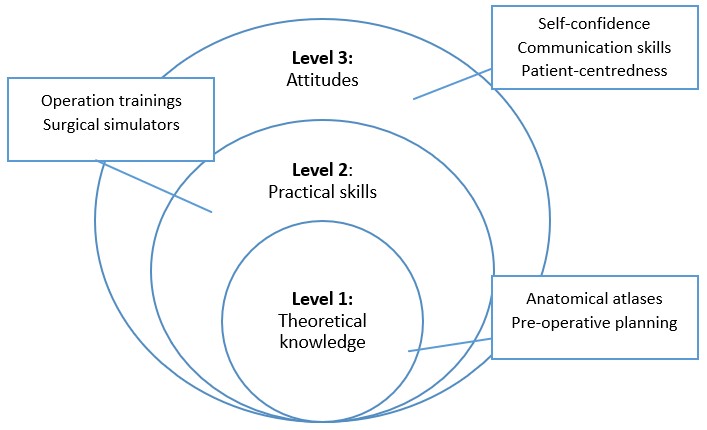


Three levels of knowledge for XR (adapted and modified from Górski et al., 2017).
