# Supplementary material for: Augmented, Mixed, and Virtual Reality-Based Head-Mounted Devices for Medical Education: Systematic Review
Source: JMIR Serious Games. 2021 Jul 8;9(3):e29080. doi: 10.2196/29080 (PMC8299342; doi:10.2196/29080)
Supplement: Multimedia Appendix 6 [file games_v9i3e29080_app6.docx]

**Multimedia Appendix 6. Number and type of study participants.**

| **Literature Reference** | **Number Study Participants (n)** | **Type of Study Participants** |
| --- | --- | --- |
| Azimi et al. (2018) [41] | 20 | residents |
| Bairamian et al. (2019) [43] | 11 | residents, physician |
| Barré et al. (2019) [2] | 10 | residents |
| Bing et al. (2019) [53] | 10 | residents |
| Butt et al. (2018) [40] | 20 | students |
| Dyer et al. (2018) [55] | 178 | students |
| Ekstrand et al. (2018) [39] | 64 | students |
| Farahani et al. (2016) [54] | 3 | physicians |
| Ferrandini et al. (2018) [38] | 67 | students, physicians |
| Harrington et al. (2018) [56] | 40 | students |
| Hooper et al. (2019) [37] | 14 | residents |
| Huang et al. (2018) [48] | 32 | residents, physicians |
| Huber et al. (2017) [44] | 10 | varying training levels |
| Leitritz et al. (2014) [36] | 37 | students |
| Lin et al. (2015) [45] | 1 | physician |
| Logishetty et al. (2019) [35] | 24 | students |
| Luursema et al. (2017) [34] | 63 | students |
| Moro et al. (2017) [50] | 20 | students |
| Peden et al. (2016) [33] | 14 | students |
| Pulijala et al. (2018) [32] | 95 | residents |
| Qin et al. (2019) [46] | 32 | residents |
| Rai et al. (2017) [31] | 28 | residents |
| Rochlen et al. (2017) [49] | 40 | students, residents |
| Siff and Mehta (2018) [52] | 18 | residents |
| Stepan et al. (2017) [51] | 66 | students |
| Wu et al. (2014) [30] | 40 | varying training levels |
| Yoganathan et al. (2018) [47] | 40 | physicians |
