# Supplementary material for: Augmented, Mixed, and Virtual Reality-Based Head-Mounted Devices for Medical Education: Systematic Review
Source: JMIR Serious Games. 2021 Jul 8;9(3):e29080. doi: 10.2196/29080 (PMC8299342; doi:10.2196/29080)
Supplement: Multimedia Appendix 7 [file games_v9i3e29080_app7.docx]

**Multimedia Appendix 7. Effectiveness as reported in the included studies for knowledge, skills, and attitude outcomes**

| **Intervention** | **Result** | | | **Medical Discipline** | **Head-Mounted Display** | | | | **Literature Reference** |
| --- | --- | --- | --- | --- | --- | --- | --- | --- | --- |
| **Effectiveness – Knowledge Outcomes** | | | | | | | | | |
| 3D Angiograms in VR Environment | **Partly effective** | | | Surgery | Google Daydream | | | | Bairamian et al. (2019) [43] |
| Neuroanatomy Training | **Additional tool*** | | | Anatomy | HTC Vive | | | | Ekstrand et al. (2018) [39] |
| Examination of Digital Pathology Slides | **Partly effective** | | | Pathology | Oculus Rift | | | | Farahani et al. (2016) [54] |
| Stereopsis and VR in Anatomy Learning | **Not proven** | | | Anatomy | Oculus Rift | | | | Luursema et al. (2017) [34] |
| Anatomical Knowledge | **Effective** | | | Anatomy | Oculus Rift / Samsung Gear VR | | | | Moro et al. (2017) [50] |
| Holographic Gynaecologic Surgery | **Effective** | | | Gynaecology | HoloLens | | | | Siff and Mehta (2018) [52] |
| Learning Neuroanatomy | **Additional tool*** | | | Anatomy | Oculus Rift | | | | Stepan et al. (2017) [51] |
| **Effectiveness – Skills Outcomes** | | | | | | | | | |
| Needle chest decompression for direct line | **Effective** | | Surgery | | | | HoloLens | | Azimi et al. (2018) [41] |
| Sleeve Gastrectomy | **Effective** | | Surgery | | | | HTC Vive | | Barré et al. (2019) [2] |
| Surgery cervical cancer | **Effective** | | Gynaecology | | | | Oculus Rift | | Bing et al. (2019) [53] |
| Urinary Catheterization | **Effective** | | Urology | | | | Samsung Gear VR | | Butt et al. (2018) [40] |
| Rapid treatment in a Mass Causality Incident | **Additional tool*** | | Emergency Medicine | | | | Samsung Gear VR | | Ferrandini et al. (2018) [38] |
| 360° operative videos | **Effective** | | Surgery | | | | Samsung Gear VR | | Harrington et al. (2018) [56] |
| VR Training in THA | **Partly effective** | | Surgery | | | | Oculus Rift | | Hooper et al. (2019) [37] |
| Central line simulation | **Effective** | | Surgery | | | | Brother AirScouter AR Glasses | | Huang et al. (2018) [48] |
| Laparoscopic surgery simulation | **Effective** (Research needed) | | Surgery | | | | HTC Vive | | Huber et al. (2017) [44] |
| Ophthalmoscopic examination | **Effective** | | Ophthalmology | | | | Eyesi Indirect System Headset | | Leitritz et al. (2014) [36] |
| Dental Implant Surgery | **Effective** | | Dentistry | | | | Sony HMZ T1 | | Lin et al. (2015) [45] |
| Accuracy of Acetabular Cup Orientation in Simulated THA | **Additional tool*** | | Surgery | | | | HoloLens | | Logishetty et al. (2019) [35] |
| Basic Surgical training | **Not effective** | | Surgery | | | | Google Glass | | Peden et al. (2016) [33] |
| Peg transfer training | **Effective** | | Surgery | | | | HTC Vive | | Qin et al. (2019) [46] |
| Binocular indirect ophthalmoscopy | **Effective** | | Ophthalmology | | | | Eyesi Bio simulator headset | | Rai et al. (2017) [31] |
| Needle insertion | **Effective** | | Surgery | | | | Epson Moverio | | Rochlen et al. (2017) [49] |
| Ultrasound in Emergency Medicine | **Partly effective** | | Emergency Medicine | | | | Google Glass | | Wu et al. (2014) [30] |
| Acquisition of knot-tying skills | **Effective** | | Surgery | | | | Not specified | | Yoganathan et al. (2018) [47] |
| **Effectiveness – Attitude Outcomes** | | | | | | | | | |
| Empathy for age- related health problems | **Effective** | Geriatrics | | | | Oculus Rift | | Dyer et al. (2018) [55] | |
| Effect of surgery on self-confidence | **Effective** | Surgery | | | | Oculus Rift | | Pulijala et al. (2018) [32] | |

*Additional tool refers to HMD being recommended only in conjunction with traditional tools.
