# Supplementary material for: Augmented, Mixed, and Virtual Reality-Based Head-Mounted Devices for Medical Education: Systematic Review
Source: JMIR Serious Games. 2021 Jul 8;9(3):e29080. doi: 10.2196/29080 (PMC8299342; doi:10.2196/29080)
Supplement: Multimedia Appendix 8 [file games_v9i3e29080_app8.docx]

**Multimedia Appendix 8. Benefits, shortcomings, and recommendations described within included studies.**

| **Recommendations (by authors of included publications)** | |
| --- | --- |
| Long-term research on effectiveness needed  IT improvements needed  Further application to low-resource context needed  More research on safety and reliability of the tool  XR feedback mechanisms needed e.g. intraoperative complications  Further studies on the potential of information retention through XR  More research on relation of previous surgical experience towards the usefulness of tool Additional research in THA before it can become part of curriculum  Implement surgical tutors who are experts in XR during trainings Understanding the specific needs of certain medical groups  Case libraries missing – one database for best practice simulation procedures Monitoring of progress missing  Only straightforward procedures – no variations in disease severity in simulation Navigation difficulties with input devices – improvements needed | |
| **Benefits (as stated by the authors of included publications)** | |
| Increased accuracy and precision  Better performance compared to conventional methods  Higher engagement  Increased motivation to learn  Learning in unsupervised setting  Increased time-efficiency  Possibility of repetitive training  Self-directed learning  Usefulness | Increased cost-efficiency  Better knowledge retention  More attentiveness  More self-confidence  Feasible education tool  Immersive training  Less head movements  Increased skills outcomes  Spend more time on training |
| **Shortcomings** | |
| As effective as traditional methods Image resolution limited  Navigation difficulties  Motion sickness  Stressful training experience  No difference in surgical accuracy Trajectory and rotation angle not recorded No improvements in surgical capability Took longer to accomplish task | No effects on information retention  Human judgement needed  Hurts on the head  Time to understand  Cannot reach dexterity of physician’s hand  Variations in disease severity not shown  No useful medical app for XR available |
